# Predictive Validation of an Influenza Spread Model

**DOI:** 10.1371/journal.pone.0065459

**Published:** 2013-06-03

**Authors:** Ayaz Hyder, David L. Buckeridge, Brian Leung

**Affiliations:** 1 Department of Biology, McGill University, Montreal, Quebec, Canada; 2 Surveillance Lab, McGill Clinical and Health Informatics, McGill University, Montreal, Quebec, Canada; 3 Department of Epidemiology, Biostatistics and Occupational Health, McGill University, Montreal, Quebec, Canada; 4 Agence de la santé et des services sociaux de Montréal, Direction de santé publique, Montreal, Quebec, Canada; 5 School of Environment, McGill University, Montreal, Quebec, Canada; National Institutes of Health, United States of America

## Abstract

**Background:**

Modeling plays a critical role in mitigating impacts of seasonal influenza epidemics. Complex simulation models are currently at the forefront of evaluating optimal mitigation strategies at multiple scales and levels of organization. Given their evaluative role, these models remain limited in their ability to predict and forecast future epidemics leading some researchers and public-health practitioners to question their usefulness. The objective of this study is to evaluate the predictive ability of an existing complex simulation model of influenza spread.

**Methods and Findings:**

We used extensive data on past epidemics to demonstrate the process of predictive validation. This involved generalizing an individual-based model for influenza spread and fitting it to laboratory-confirmed influenza infection data from a single observed epidemic (1998–1999). Next, we used the fitted model and modified two of its parameters based on data on real-world perturbations (vaccination coverage by age group and strain type). Simulating epidemics under these changes allowed us to estimate the deviation/error between the expected epidemic curve under perturbation and observed epidemics taking place from 1999 to 2006. Our model was able to forecast absolute intensity and epidemic peak week several weeks earlier with reasonable reliability and depended on the method of forecasting-static or dynamic.

**Conclusions:**

Good predictive ability of influenza epidemics is critical for implementing mitigation strategies in an effective and timely manner. Through the process of predictive validation applied to a current complex simulation model of influenza spread, we provided users of the model (e.g. public-health officials and policy-makers) with quantitative metrics and practical recommendations on mitigating impacts of seasonal influenza epidemics. This methodology may be applied to other models of communicable infectious diseases to test and potentially improve their predictive ability.

## Introduction

The influenza virus presents several challenges for society. These challenges are preventable mortality and morbidity in vulnerable populations (e.g. infants, seniors, those with chronic conditions), and social, economic and health care costs during pandemics and seasonal epidemics. Effective interventions against influenza include vaccination and social distancing. Another tool which policy-makers have at their disposal, for mitigating these impacts, is mathematical and computational models [1]–[3]. Models help policy-makers to design, implement and evaluate effective and practical mitigation strategies. In this process, model predictions are directly linked to the decisions of policy-makers. In turn, these decisions benefit society when they work but may also have significant costs associated with their failure to mitigate impacts. Given the potential for severe epidemics, the seasonal nature of influenza epidemics and the intimate links between modeling and policy, we must critically and continually evaluate predictions of influenza spread models in the context of their use by policy-makers.

As an example of how policy makers use model predictions, consider how they plan for the influenza season. At the start of the season, policy-makers need to decide on matters such as the number of vaccines to order from pharmaceutical companies and optimal allocation of vaccines among health-care workers (HCWs), community and other vulnerable populations. Apart from vaccination, policy-makers may also use strategies of social distancing, quarantine, school closures or a combination of several strategies [4]. Model predictions regarding how epidemics will spread under each mitigation strategy are captured by epidemic metrics such as absolute intensity, peak week and epidemic duration [5]–[9]. Predictions of epidemic intensity guide policy decisions on vaccine production, distribution, and stockpiling. Predictions of epidemic timing, such as when the epidemic will start, peak and end, aid public-health officials in deciding when to start and complete vaccination, education and awareness campaigns in high-transmission setting such as schools, hospitals and long-term care facilities. Predictions about epidemic duration are useful for designing mitigation strategies that are sustainable and remain effective throughout the epidemic period. Policy-makers estimate these and other metrics based on predictions from forecast models under different mitigation strategies. Optimal strategies are then used to develop policy in the hope that the predicted reduction in impacts will be realized in the real-world setting.

Although models can inform policy-makers in several ways, we argue that their predictive accuracy should be validated. Unreliable predictions of epidemic metrics such as peak week, intensity and duration have far-reaching consequences for society including higher than expected economic and health-care costs if models underestimate epidemic intensity and insufficient mobilization of resources if the epidemic lasts longer than expected [10]. Also, planning for an epidemic which actually peaks later or lasts longer than expected may also lead to logistical challenges. For example, resources and effort budgeted for a specific time-frame may not be easily extended if the epidemic last longer than expected by forecast models of the ongoing epidemic. Model-based forecast of future epidemics is a critical piece of information in planning for influenza and other infectious diseases [11], [12]. Given these uncertainties in epidemic metrics and their practical use in making critical policy decisions, it is imperative to validate the predictions of current models of influenza spread.

Model validation is included in current models of influenza spread but may require a different approach when model predictions are used as inputs for policy-making. The main benefit of validating a model is greater confidence in its capabilities and output, but also the revision of its assumptions when predictions do not match observed data. Current approaches towards model validation and assessment of predictability include i) graphical evidence of a good match between the simulated and observed epidemic trajectory [13]–[16] or number of cases [17], ii) analysis of parameter uncertainty and sensitivity [11], [18]–[20] and iii) analysis of model associated uncertainty [4], [21]. Others have also attempted to relate connections in the airline traffic network [22], vitamin D exposure [23] and absolute humidity [24] as predictors of influenza. These validation techniques primarily address conceptual and operational validity, but we argue that validation needs to go beyond the current view that fitting the model to a single epidemic is sufficient to show the predictive validity of the model.

For our purposes, we define predictive validation as a process that explores the deviation between observed and predicted patterns under the assumption that the processes underlying the model are generalizable. Practically, this assumption means we should be able to perturb the system (e.g. different vaccination strategies), measure and include the perturbation in the model, and still reliably forecast new, different patterns (e.g. predict the consequences of alternative vaccination regimes). We believe that the ability of models to predict future epidemics not used to build the model serves as a more robust test of predictive validation than model-fitting. There may be consequences for basing decisions on non-predictive models. Arguably, it is optimal to identify such deviations now, when time exists to explore and improve current models, rather than during a crisis when there may not be the luxury of rigorous analysis. Well-developed techniques for the validation of simulation models exist [25]–[27], yet little work has been done to specifically address the predictive validity of current simulation models for disease spread. One study that we are familiar with looked at the predictive ability of the 2009 H1N1 Pandemic after matching only the first wave of the pandemic [28]. This is very different from the definition of predictive validation we are proposing because it does not address the idea of perturbations. Furthermore, our study is specifically looking at several seasonal epidemics whereas the Merler et al. study looked specifically at pandemics.

We focus on individual-based models (IBMs), which are a class of simulation models that policy-makers are turning to for guidance on epidemic and pandemic planning [21]. In an IBM, the attributes, behaviors and interactions of individuals are modeled through complex, non-linear, feedback and adaptive processes. These are attractive features, which policy-makers may want a spread model of influenza to consider, because the greater degree of model realism leads to better understanding of transmission dynamics and the application/testing of novel mitigation strategies. The efforts of the Models of Infection Disease Agent Study (MIDAS) are a prime example of the efforts underway to use simulation models of varying complexity to design better mitigation and control strategies, guide policy decisions and reduce the burden of influenza, among other disease, on society. Although simpler models suffice for real-time forecasting of epidemics, they are limited in several ways. First, their ability to evaluate the effectiveness of novel mitigation strategies is limited by their compartmental-model like formulation. Second, they are not as useful for understanding the complex interactions between host, disease and environment. Lastly, policy makers continue to take interest in more complex models, as evidenced by the MIDAS effort. Therefore, we argue that as long as policy makers are going to make use of IBMs we should evaluate their predictive ability. In addition to these reasons, disease transmission models for influenza and other communicable diseases continue to be integrated with climate models to make predictions about the burden of illness under a changing climate [29]. This further shows that it is timely to investigate the predictive ability of complex simulation models. Also, recently several authors have use the IBM approach to model the spread of influenza at the city-scale [4], [14], [15], [30], [31] and country-scale [11], [13], [19], [32]. The rapid development and application of IBMs, over the last few years, for setting policy and making decisions makes them an ideal candidate for model validation with an emphasis on predictive ability.

In brief, the purpose of our study is to examine the process of predictive validation using a current and well-known IBM for the spread of influenza rather than to formulate a model that makes good predictions. These are two very different purposes and we only focus on the first one. Briefly, we will generalize the IBM to our study area and modify it to include perturbations of interest, estimate these perturbations from the real world and include them in the model, and measure the deviations between observed and simulated epidemics. The novelty of our study lies in two features. First, we will validate the model based on data from several observed epidemics on which we have detailed laboratory-confirmed data. Previous studies using this IBM had limited access to empirical data (e.g. one or a few seasons only). Therefore, these studies simply showed that the model was able to do a good job of predicting a single epidemic [13], [14], [19]. Our approach is a better test of predictive validation because models may more easily be calibrated to coincide with a single epidemic than capture new, different epidemic patterns. Second, our process of predictive validation utilizes perturbation factors (influenza strain and vaccination coverage in the system) which have immediate and practical implications for epidemic planning. Our study provides useful guidance, since model predictions, for influenza but also other communicable diseases, serve as important knowledge inputs into policy- and decision-making during times of public-health crises.

## Methods

The process of predictive validation requires the integration of model and data in novel ways. As an outline of our methods, we first described the IBM of Ferguson et al. [11] and how we generalized their model to the urban setting of Montreal, Quebec, Canada. Then, we provided a description of our data on past epidemics and the derivation of different epidemic metrics. The process of predictive validation, which we will describe in detail, brought together our generalized model, real-world data and epidemic metrics to provide a qualitative assessment of model’s predictive ability to forecast future epidemics.

### Montreal IBM Model

For our study, we chose to generalize a well-known IBM for the spread of influenza. The IBM was originally developed by Ferguson and colleagues in two papers [11], [19]. Both of these studies have been cited over 900 times since publication. The basic modeling approach of Ferguson et al. [11] has been generalized to various geographic settings such as cities (Buffalo, NY [14] and Chicago, IL [4] in the United States) and countries (Italy [32] and Switzerland [13]). Their model, which we will refer to as the Ferguson IBM, integrated some of the most relevant demographic, institutional (e.g. schools and workplaces), and disease natural history variables into a spatially-explicit stochastic simulation model. More concretely, the Ferguson IBM included data on age, household size and place size (workplace and schools), a time-dependent infectiousness profile, and random community contact. We generalized the Ferguson IBM to the Census Metropolitan Area of Montreal (CMA Montreal). We referred to our generalized model as the Montreal IBM. We chose this spatial scale for two reasons. First, we had extensive data on several past seasonal influenza epidemics at this scale. Second, municipal public-health departments not only form a critical part of the influenza surveillance network, but also have direct responsibility for implementing mitigation strategies. Therefore, modeling at the city-scale was relevant and practical for forecasting epidemics.

Given our study’s focus on determining the predictive ability of the Montreal IBM, we provided limited details on specific model parameters that were previously estimated by Ferguson et al. from the literature. Examples of such parameters included, but not limited to, the infectiousness profile function, latent period, and specific components of force of infection. We had two reasons for not discussing these modeling details as well as their sensitivity analyses. First, these parameters were not likely to change for our study area (a modern North American city) since they were previously estimated and applied by Ferguson et al. [11] to the United States and Great Britain. Second, other studies, which have been based on the model of Ferguson et al. [11], have not challenged, for the most part, the assumptions and functional form of the disease transmission process underlying the model. Consequently, we provided details on only those model components and parameters that were needed to generalize the Ferguson IBM to CMA Montreal, in turn, producing the Montreal IBM. Unless indicated, we left unchanged modeling assumptions in Ferguson et al. [11]. The specific components of the Ferguson IBM, which we modified, included re-estimating the transmission coefficients and modifying assumptions regarding pre-existing immunity. Details of these changes are given in the model description below.

### Disease Transmission Model

We modeled the disease transmission process following the work of Ferguson et al. [11], [19]. In their model, a force of infection (*λ_i_*) was calculated for each individual *i* at each time *t*. There were three sources of infectious contact that influenced the force of infection. They were household, place (work/school) and random community contact. We assumed that 30% of all transmission occurred in the household. The transmission parameters were chosen to match various characteristics of influenza epidemics: i) R the effective reproduction number (from the literature) ii) age-based clinical attack rates (from literature and hospital utilization data for our study area), and iii) fit of the simulated epidemic curve to an observed epidemics trajectory (from laboratory confirmed data for the 1998–1999 season). R was calculated by the equation *1* *+rT*, where *r* is the growth rate of the simulated epidemic and T is the generation time estimated from the infectiousness profile function. We estimated *r* from the stable region of the epidemic growth curves and T was set to 2.6 and 3.2 days in epidemics under the general and specific strain infectiousness profile function. For these values of T, we estimated r between generations 6 and 9, inclusive, for the general strain and between generations 5 and 7, inclusive, for the specific strain. We discuss differences between these infectiousness functions later as it was one of the perturbation variables we considered in our study. Data on age-based clinical attack rates were from the literature and estimated from hospital utilization data for influenza-like-illness (ILI) diagnosis. The ILI data were from in-patient, out-patient and emergency hospital settings for CMA Montreal from 1996–2006. Although different studies have reported clinical attack rates using different categorization of age years, we primarily used the categories 0–17 and 18+ (i.e. 18 and above) to qualitatively determine the match between the simulated and observed clinical attack rates. In the supporting information material (Text S1), we also provided clinical attack rates by different age categories. Lastly, we used the fit of the simulated epidemics to the observed data on laboratory-confirmed counts of cases testing positive for influenza virus. Details on these data are given in the next section. Using these three different methodologies, we estimated the transmission factors for the general and specific strain scenarios (Table 1). We scaled the school transmission coefficients to match the observed patterns in clinical attack rates. These transmission coefficients were used to multiply the different infectiousness profiles, which were scaled to a maximum value of 1 (Fig. S3). Due to differences in the shape of these profile functions, different transmission coefficients were needed to match the simulated epidemics to expected patterns. Our primary aim here was to generate reasonable approximations to real-world epidemics so that we could use the fitted epidemic in the predictive validation exercise. Although the Montreal IBM is probably able to also match season-to-season dynamics, this was beyond the scope of our study because this study’s aim is to evaluate predictive ability of the model after the start of each future season rather than season-to-season dynamics. Using these transmission coefficients, we calculated the force of infection as, 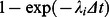, for each individual. We assumed *Δt* = 0.25 days in line with Ferguson et al. [19]. Further details on the formulation of the Montreal IBM are provided in the Text S1.

**Table 1:** Best-fit values of important model parameters in baseline models fitted to reference influenza season (1998–1999) and values for fit metric, R and age-based clinical attack rates based on 50 simulated epidemics under the best-fit parameters.

Infectiousness profile in baseline model	Scalar forpre-existing immunity	Transmission coefficients				Scalar for simulated epidemiccurve (θ)	R (min, max)from simulatedepidemics	Fit metric(D)*	Clinical Attack Rates (age-based)	
		Household	School	Workplace	Community				0–17	18+
**General**	0.25	0.50	4.39	0.55	0.06	3.66E-004	1.71 (1.51, 1.81)	4.27	27%	5%
	0.5	0.47	4.12	0.52	0.05	3.08E-004	1.70 (1.60,1.82)	4.07	27%	5%
**Specific**	0.25	0.78	6.92	0.86	0.09	1.08E-004	1.81 (1.60, 1.93)	4.09	34%	17%
	0.5	0.89	7.85	0.98	0.10	1.32E-004	1.97 (1.80, 2.06)	4.37	37%	24%

Results are presented for both infectiousness profile functions and assumptions for pre-existing immunity.

* Lower value indicates better fit.

### Modeling Pre-existing Immunity

To model pre-existing immunity, we assumed that a proportion of the population was naturally immune due to vaccination in previous years with a certain level of vaccine effectiveness. We also assumed that individuals were vaccinated at the start of each season. Given the considerable variation that exists in estimates of natural immunity, we presented all efforts at predictive validation under two levels of natural immunity. Methodologically, we did this by scaling the force of infection by 0.25 or 0.5. The practical implication of this assumption is that individuals are somewhat protected against infection but not completely. To model the idea of vaccine effectiveness, we further assumed that the probability of infection for each individual was reduced by a certain percentage based on their age. The reductions were varied to reflect that the older population was more protected than the younger population (i.e. 0% for 0–2 yrs. old, 50% for 3–64 yrs. old and 20% for 65 years and above. To model vaccination efforts at the start of the influenza season, we assumed similar levels of vaccine effectiveness each season and assumed a proportion of the population was vaccinated but never fully protected against infection. These proportions were estimated from published data from government reports on vaccination efforts and are further described later as vaccination coverage was a perturbation variable in this study.

### Data on Past Epidemics and Perturbations

Our choice of data, epidemic metrics and the two perturbation variables, was motivated by the following considerations, i) model predictions should be validated in the context of their intended use and application in the real-world, and ii) policy-makers consider them important in epidemic planning. We used laboratory-confirmed data to estimate various epidemic metrics (peak week, absolute intensity, epidemic duration).

#### Laboratory-confirmed data on influenza

We used laboratory-confirmed viral testing data to fit the baseline model. We used the baseline model to retrospectively simulate “future” epidemics and ate epidemic metrics. The laboratory data were obtained from the Laboratoire de Sante Publique du Quebec (Quebec Public Health Laboratory) for the years 1998 to 2006, inclusive (Table 2). These data contained the number of samples tested and percentage of positive samples for each week of laboratory disease surveillance. Using these data, we calculated the number of positive samples to characterize the trajectory of the observed epidemic in each season. Given that reporting bias is common among these types of data, we reported both the number and % of laboratory positive viral samples but used only the former to fit the baseline model and forecast future epidemics. Although these data were aggregated at the provincial level, we assumed that they provided a good approximation of the expected epidemic patterns in our study area. This was a reasonable assumption since our study area comprised of approximately 53% of the population of Quebec. As well, Quebec City was the only other major urban center in the province of Quebec and its close geographic proximity to CMA Montreal (250 km) meant that its population, more or less, experienced seasonal influenza epidemics with similar temporal characteristics.

**Table 2:** Epidemic metrics based on laboratory-confirmed samples positive for influenza virus from the Laboratoire de Sante Publique du Quebec (Quebec Public Health Laboratory).

Flu season	Start week of epidemic*	End week of epidemic*	Peakweek	Absolute intensity(number ofpositive samples)	Epidemicduration (weeks)	Predominant-circulatingtype of influenza virus
1998–1999	51	14	9	61	16	A
1999–2000	43	13	13	40	23	A
2000–2001	51	14	11	66	16	B
2001–2002	48	14	10	73	19	A
2002–2003	50	18	11	28	21	A
2003–2004	47	13	12	53	19	A
2004–2005	45	15	9	94	23	A
2005–2006	4	20	12	46	17	A

* Calendar weeks.

#### Epidemic metrics

We used our laboratory data to derive various epidemic metrics such as peak week, absolute intensity and epidemic duration. These metrics described different characteristics of the epidemic such as timing and intensity. Peak week was calculated as the week with the highest number of positive culture samples and absolute intensity was the number of samples in the peak week. Epidemic duration was slightly more difficult to define. Different authors have used different definitions of epidemic duration for seasonal influenza [8], [33], [34]. Chan et al. [33] defined the start of the epidemic as approximately 4 weeks before the first two consecutive weeks in which the number of positive samples was equal to or greater than 5 in both weeks. Their approximation of 4 weeks was a conservative estimate since the epidemic was definitely underway after this time, but there were also time lags to consider, depending on the source of surveillance data. The end week of the epidemic was defined as one week before observing less than 5 positive cases. Rather than constructing our own definition of epidemic duration, we opted for the definition of Chan et al. [33]. We calculated these three epidemic metrics for each influenza season (Table 2).

### Perturbation Variable I: Vaccination Coverage

Annual variation in vaccination coverage was a natural choice for a perturbation. Vaccination has long been an important mitigation strategy for seasonal influenza. We used annual reports on vaccination in Quebec, in Canada, and other published data to determine the percentage vaccination coverage in each of the following age categories: infants (0–2 yrs. old), kids (3–18 years old), adults (19–64 years old) and seniors (65 years and older) (Table 3). The most useful data on vaccination coverage was available for infants and seniors, since they are highly susceptible to influenza infection. For a few seasons, data were not available in some groups such as adults and infants. For age groups in these seasons, we interpolated values from other seasons for which data were available (Table 3). We did not find any other data for the kids’ category, since most of the literature reported vaccination coverage rates only for those in this age group who had one or more chronic condition. Since kids and adults were normally not considered as high-risk groups for infection, we interpolated their vaccination coverage values. We assumed that kids had an average level of coverage between infants and adults.

**Table 3:** Vaccination coverage levels by age categories [minimum age, maximum age].

Flu season	Infants [0.5–2]	Kids [3]–[17] *	Adults [18–64]	Seniors [65–100]	Coverage in overall population (simulated)
1996–1997	12%†	9%	7%§	42%	14%
1997–1998	13%†	10%	7%§	46%	15%
1998–1999	15%†	12%	8%§	44%	16%
1999–2000	17%†	13%	9%§	52%	19%
2000–2001	20%†	15%	10%	59%	22%
2001–2002	22%†	17%	11%§	57%	23%
2002–2003	26%†	18%	11%	59%	24%
2003–2004	29%	21%	13%§	61%	27%
2004–2005	34%	24%	14%§	65%¶	30%
2005–2006	38%	27%	16%	68%¶	34%

* Coverage values are assumed to be the average of values for Infants and Adults.

† fitted value based on 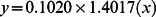, where *x* = integer index of the season, same definition in equations for fitted values of coverage below.

§ fitted value based on 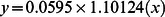.

¶ fitted value based on 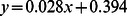.

### Perturbation Variable II: Influenza Strain Data

It is well-known that antigenic shifts in the influenza virus’s RNA may lead to differences in virulence and transmission between each season. Also, policy-makers may have knowledge about the most likely influenza strain which may show up in their administrative region. Such knowledge may be assembled from global influenza surveillance programs (e.g. WHO) or other nearby regions where the epidemic may already be underway. It may come to pass that policy-makers may be uncertain about the information they have received on the influenza strain for a given season. Therefore, when faced with decisions, which must be made in real-time, policy-makers may want to know how uncertainty in strain information translates through to various epidemic metrics and the overall epidemic trajectory.

One way to do this may be through forecasting under functions that capture the general or strain-specific infectiousness of the influenza virus. Data on the influenza strain was available from several sources for our specific study area and time period. Our laboratory-confirmed data provided a breakdown of positive samples by influenza type (A or B) but not subtype. Also, Chan et al. [33] reported the predominant circulating strain by type and subtype from 1998 to 2003, inclusive, in the province of Quebec. This latter data source was used to confirm the predominant circulating strain of influenza for each season according to the laboratory-confirmed data (Table 2). In order to incorporate strain-specific data in the Montreal IBM, we used the infectiousness profile functions reported by Liao et al. [35] (scaled to a maximum value of 1, Fig. S1). These functions were estimated based on experimental data on infections for each of the following strains, type A (H1N1), type A (H3N2) and type B influenza. We used the infectiousness profile function for type A (H1N1) because the same strain was the predominant circulating strain in a majority of the seasons for which Chan et al. [33] had reported subtype-level data. The 2000–2001 season was dominated by the influenza B type strain. We excluded this season from our analysis because we required two seasons with type B as the predominant strain where one season was used to fit the model and another season to forecast from the fitted model. In effect, the perturbation we were concerned with was not which specific strain will circulate during a season, but instead which infectiousness profile function to use, general or strain-specific, in a forecast model when there was uncertainty about which strain was likely to emerge for a future season. The general strain infectiousness profile function we used referred to the original function in Ferguson et al. [19]. In practice, if policy-makers had good information on the strain, then it was possible to use the strain-specific profile to forecast the upcoming epidemic.

### Predictive Validation

The process of predictive validation involved three steps i) establishing a baseline model, ii) perturbing the baseline model to forecast epidemics and iii) quantifying the differences in epidemic patterns between simulated (forecast) and observed epidemics. We want to reiterate here that we are not trying to capture season-to-season dynamics but rather how well the Montreal IBM would have predicted each future influenza season using the baseline model fitted to the reference season.

#### Baseline model

The purpose of the baseline model was to describe an observed epidemic known to have taken place under a set of real-world perturbations. Our rationale was that if a model had good predictive ability, then policy-makers, when provided with this baseline model, should be able to forecast future epidemics by only changing the perturbation variables and nothing else. By using the baseline model in this manner, we assumed that the model sufficiently captured the processes underlying the spatial spread of influenza. There was good reason to make this assumption, since the basic model of Ferguson et al. [11] has not been modified dramatically in any of the other studies which have used it in different geographic locations and spatial scales. This implied trust and confidence in the underlying processes of the model.

We evaluated the predictive validity of the Montreal IBM by establishing a baseline model that was fitted to laboratory-confirmed data on seasonal influenza in 1998–1999. We chose this reference season based on a previous analysis by Chan et al. [33]. They reported the highest correlation in time series data from various sources for this season over others in the same study area as ours. We took this to mean that the timing, peak and duration of the 1998–1999 influenza epidemic was most likely to have been represented well by the various types of data, including laboratory confirmed data. In other words, reporting bias was probably lower in the laboratory data for this season than other seasons for which we had available data.

IBMs are very complex models for which fitting each parameter takes considerable computational resources. We estimated the transmission coefficients, as mentioned above, and vaccinated individuals in the baseline model according to the observed data on coverage levels by age group for the reference season (Table 1). In these simulations, we also modeled all previously stated assumptions regarding pre-existing immunity and vaccine effectiveness. Due to the differences in the estimation of the infectiousness profiles and our assumptions about pre-existing immunity, we fitted baseline models under four scenarios, which are described by the first two columns in Table 1. For the general strain, we used the infectiousness profile based on Ferguson et al. [11]. For the specific strain, we used the infectiousness profile function based on Liao et al. [35] and discussed above.

To estimate the fit of the model, we used a modified version of the percent error function (denoted by *D*) which minimized the difference between the observed and simulated epidemic.
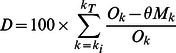
(1)


*O_k_*, was the number of positive laboratory-confirmed viral samples in week *k* since the start of the epidemic.The epidemic ended by week *k_T_*. *M_k_(γ)* was the number of infected individuals in week *k* of simulated epidemics. Since the simulated epidemic curve indicated the total number of new infections at each time step, we applied a scalar, θ, to make the simulation results comparable to the actual number of laboratory-confirmed positive viral isolates. Since there was no way to know this scaling factor, we had to estimate it simultaneously with each different value of the transmission coefficients.

We simulated all epidemics by randomly infecting 100 individuals. This was done to ensure that epidemics actually took place. Also, to ensure that we were not picking from very rural areas, we only individuals to be initially infected were only chosen if they lived in grid cells with ≥100 individuals. This ensured that a large number of epidemics did not die out. To calculate *D*, for any given value of θ, we first simulated epidemics (N = 50) under various values of the transmission coefficients. We limited the number of simulations to 50 due to the considerable computational resources required in IBMs. Next, for each simulated epidemic curve we estimated a value of *θ* which minimized *D* (Eqn. 1). We explored a wide range of values for θ [1e-2, 1e-6] with a step size of 1e-6 using the grid search method. Prior to calculating *D*, we shifted each epidemic curve (scaled by θ) in time to match the start week of the epidemic in the reference season. We matched the starting weeks in the observed and simulated epidemic curve primarily because, i) we do not know the exact date when the epidemic started in the study area, and ii) there may be uncertainty in our assumptions about the start week of the epidemic. The only piece of information we were sure of was that the epidemic was underway after observing two consecutive weeks of 5 or more positive viral cultures in the laboratory data.

#### Predicting epidemic metrics

We predicted epidemics for each season in our study through forecast models. A forecast model was essentially the same as the fitted baseline model except that we perturbed vaccination coverage levels for the season of interest (Table 3). Since we fitted the baseline models for each type of infectiousness profile, general and strain-specific, we could make forecasts for a season under each infectiousness profile function. By defining forecast models in this way, we simultaneously accounted for annual perturbations in vaccination coverage and uncertainty about the influenza strain. Assuming that the Montreal IBM properly captured important mechanisms underlying the epidemic process, we believe this was a reasonable approach. Although policy-makers may be interested in other drivers of annual variation in epidemic patterns, these two perturbations sufficed for the purpose of our study.

To determine which parameters, others than these perturbation variables, to update in the forecast model, we turned to past studies on real-time forecasting for guidance even though they were based on influenza pandemics. These studies have primarily used simpler disease spread models. Hall et al. [6] updated the parameters of a SIR model through regression techniques to make step-ahead forecasts during an ongoing epidemic. Of the parameters they repeatedly estimated as the epidemic went on was the proportion of cases in the simulated epidemic curve represented in the observed data. This was analogous to our usage of the scalar θ, and, therefore, a good candidate for updating in our study as well. In another study, Nishiura [7] used a chain-binomial transmission model with maximum likelihood methods to forecast the 2009 H1N1 pandemic. A common assumption in both these forecasting studies was to hold constant natural history parameters of the influenza virus, such as infectiousness, transmission rate, and generation time. There were likely good reasons for this which may be equally if not more relevant for our more complex individual-based model of influenza spread. Therefore, we forecast epidemics through simpler methods using existing parameters in the model and without re-fitting parameters or modifying assumptions about the disease transmission process in the forecast model for each season.

Based on the forecast model, for any given season, we simulated epidemics (N = 50) with initial conditions similar to those we had assumed when fitting the baseline model. Each simulated epidemic curve was used to calculate several epidemic metrics such as peak week, absolute intensity and epidemic duration. This further allowed us to place error bounds on the deviation in these metrics from the data on observed epidemics. Before calculating these metrics, we scaled the simulated epidemic curve to make it comparable to the observed data. Furthermore, we matched the scaled, simulated epidemic curve to the starting week the actual epidemic, as defined previously, for that given season. In reality though, the scalar (θ), which we applied to the simulated epidemic curve, may or may not vary between each influenza season due to uncertainty in its true value. In the absence of data, which would allow for an accurate estimate this value, we assumed that policy-makers would implement a method of forecasting we called static forecasting. In static forecasting, we assumed that the estimate of θ, which was obtained from fitting the baseline model (Table 3), was the best-available estimate to policy makers. Practically, this assumption would be analogous to predicting the epidemic pattern for a future season based on some projected vaccination strategy (i.e. via a forecast model), using only current information from a past epidemic. Therefore, epidemic metrics under static forecasting were calculated by scaling each season’s forecast (i.e. the simulated epidemic curve) by the same estimate of θ as obtained for the reference season. Alternatively, policy-makers may want to re-estimate θ for each new season and possibly update its estimate as new data was gathered through laboratory surveillance by public health departments. A policy-maker’s rationale for doing so may be that since the estimate of θ would likely vary between each season then as more data became available the updated estimate of θ would likely be more accurate for the current season. We called this method of forecasting, where updating of θ took place, dynamic forecasting. In other words, for a given season, the forecast (i.e. the simulated epidemic curve) was re-scaled with each additional week of data on an ongoing epidemic. This resulted in new weekly estimates of epidemic metrics for each season. By quantifying deviations between these weekly estimates (dynamic forecasting) or fixed estimates (static forecasting) and the observed epidemic metric value for each season we were able to evaluate the timeliness and reliability of the forecast model. The ability to perform such an evaluation, under two methods of forecasting, provided a novel way to evaluate a model’s predictive ability which has not been considered, to our knowledge, in previous studies.

#### Quantifying deviations

We formulated several deviation metrics to assess the predictive ability of the Montreal IBM. Keeping in mind the relevance of model predictions for policy-makers, we evaluated each metric under assumptions for dynamic and static forecasting. One advantage of static forecasting, from a policy-makers perspective, was the availability of estimates for epidemic metrics at the very early phase of the influenza season. On the other hand, estimates under dynamic forecasting were not likely to stabilize until after observing a few more weeks of the current season given the highly stochastic early phase of influenza epidemics. To visualize the fit between the observed and simulated epidemics, under static forecasting, we plotted epidemic curves for each season. For dynamic forecasts, similar plots would have been too cumbersome to present since each week in each season produced a new epidemic curve. Instead, we plotted deviation metrics for each week of additional data, which we labeled as week of prediction in our graphs. For comparison, we also plotted the deviation metric value under static forecasting in these plots for deviation metrics.

The first metric we calculated was the error in overall fit. The purpose of this metric was to demonstrate how well the forecast model fit the overall observed epidemic. For this metric, we calculated the percent error between the observed and simulated epidemic curve. Under static forecasting, this resulted in a single value. Under dynamic forecasting, the percent error was re-calculated for each week of prediction. We expected a better match between the observed and simulated epidemic (i.e. lower % error) as additional weeks of the observed data were used in estimating θ.

The next three metrics provided a more detailed evaluation of the forecast models’ ability to predict several temporal characteristics of seasonal influenza. For peak week and epidemic duration, the deviation metric was the difference of the metric value between the forecast and observed epidemic curve. For interpretation purposes, negative and positive values in this metric meant the forecast model underestimated or overestimate, respectively, the observed epidemic metric value. For absolute intensity, the deviation metric was the percent error between the estimated value from the forecast and observed data. Absolute intensity and peak week were calculated at the actual peak week and, therefore, forecasts of these metrics only made sense until the actual peak week in each season. Conversely, epidemic duration was not dependent on the actual peak week and, therefore, we calculated its deviation metric until the end of each season.

## Results

### Statistical Properties in Observed Data

The analysis of the observed data on past epidemics revealed that there was little variation in the peak week (range of 9 to 13 weeks) and slightly more variation in epidemic duration (range of 16 to 23 weeks) (Table 2). The highest amount of variation was observed in the absolute intensity of past epidemics which ranged from 28 to 90 laboratory positive cases. Laboratory-confirmed data on past epidemics indicated that all except one influenza season were characterized by the influenza A type strain (Table 2). For about half the seasons, the timing of the epidemic (i.e. peak week) matched when we plotted the number and % of positive samples. When this was not case we observed a 1 or 2 week lag in the two laboratory-based time series data. Data on vaccination coverage levels showed different patterns across age groups (Table 3). These graphs matched the expected patterns that influenza infections are usually higher among the under 18 age group, fairly stable among adults and lowest overall among seniors. Data on vaccination coverage levels showed different patterns across age groups. We found that vaccination coverage was highest among seniors, followed by infants and then children/adults (Table 3). We also observed an overall increasing trend in vaccine coverage over the study period for all age groups and subsequently in the overall simulated population for each season.

### Predictive Validation Results

The results of the predictive validation process allowed us to evaluate the overall predictive ability of the Montreal IBM. Later, we discuss the consequences of these results on planning for influenza epidemics and provide guidance on how to improve predictions from complex simulation models of disease spread. We evaluated the predictive ability of the model through various deviation metrics and methods of forecasting. In the next section of our results, we compared the overall fit of the forecasts to the observed data under static and dynamic forecasting. In the remaining sections, we highlighted deviations between observed and forecast metric value for three different epidemic metrics by method of forecasting. In these latter sections, we focused primarily on the reliability and timing of the forecast for each epidemic metric. We preceded these sections with a few observations from fitting the baseline models.

### Model Fitting of Baseline Epidemic

**Figure 1:**
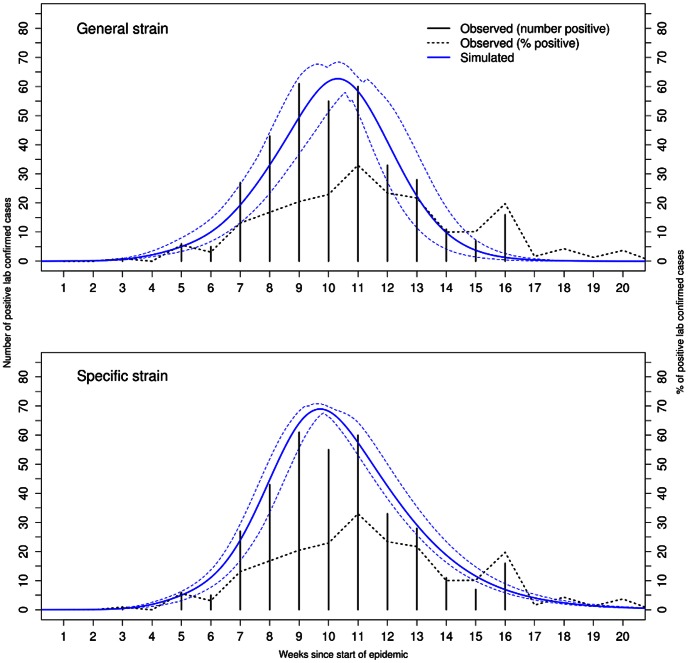
Model fitting to observed data for reference influenza season (1998–1999) under different infectiousness profile functions and scalar for pre-existing immunity set to 0.5. Observed data are presented as the number (black solid lines) and % positive (dashed black line) of laboratory-confirmed positive samples. X-axis is the number of weeks since start of season and not calendar weeks. The start of the influenza season was defined by 5 or more positive viral cultures in two consecutive weeks. Number of laboratory-confirmed samples was used to fit the simulated epidemic curve after scaling the average number of new infections (solid blue line) to compare with observed data. We matched this scaled version of the simulated epidemic curve to the first week of the actual epidemic. 95% confidence intervals were based on 50 simulated epidemics (dotted blue lines).

**Figure 2:**
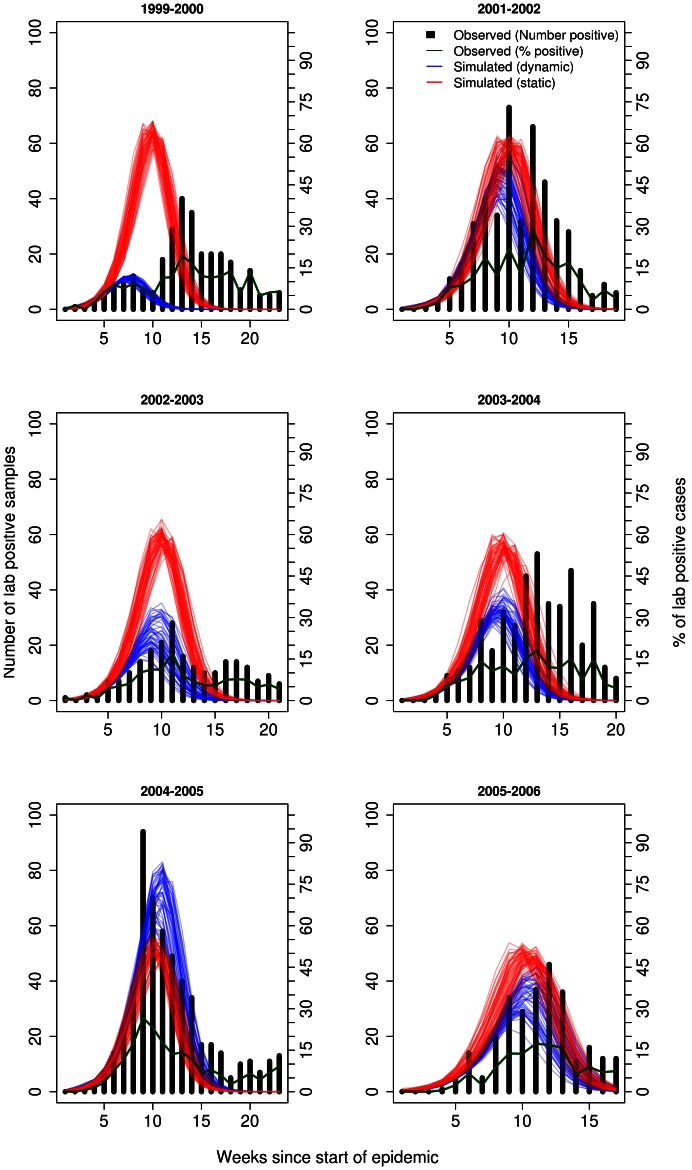
Forecasts of several past influenza seasons and the observed data on past epidemics (black bars, number of laboratory positive samples), assuming a general infectiousness profile function and scalar for pre-existing immunity of 0.50. Forecasts were based on best-fit baseline model in which only the level of vaccination coverage was changed for each season. Scaling of the simulated epidemics was done under static (based on scalar for 1998–1999 season, red lines) or dynamic (based on having observed entire epidemic for respective season, blue lines). Green lines indicated %positive laboratory samples. X-axis is the number of weeks since start of season.

**Figure 3:**
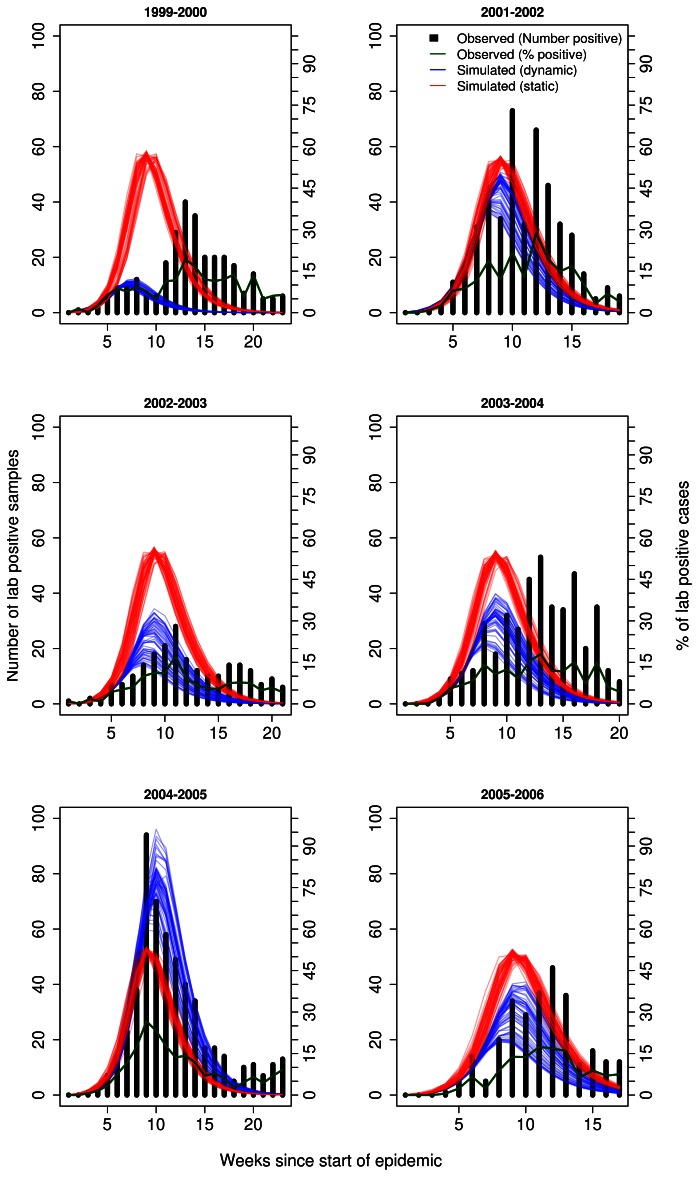
Forecasts of several past influenza seasons and the observed data on past epidemics (black bars, number of laboratory positive samples), assuming a specific infectiousness profile function and scalar for pre-existing immunity of 0.50. Forecasts were based on best-fit baseline model in which only the level of vaccination coverage was changed for each season. Scaling of the simulated epidemics was done under static (based on scalar for 1998–1999 season, red lines) or dynamic (based on having observed entire epidemic for respective season, blue lines). Green lines indicated %positive laboratory samples. X-axis is the number of weeks since start of season.

Model fitting revealed reasonable fit of the baseline model under the general and specific strain infectiousness profile to the 1998–1999 influenza season under both assumptions regarding pre-existing immunity (Fig. 1 and Fig. S2). The only noticeable exception to this finding was the fit of the simulated epidemic to observed data under the specific strain infectiousness profile when the scalar for pre-existing immunity was 0.25 (Fig. S2). Here the simulated epidemic curve underestimated the intensity of the epidemic and the confidence intervals did not encompass the observed data very well. Comparing the fit of the baseline model under the two assumed values for pre-existing immunity, we observed a better fit under value of 0.5 (Fig. 1, Table 1). A noticeable difference in the simulated epidemics under each infectiousness profile was wider 95% CI in the model for the general infectiousness profile. Using these baseline models, simulated epidemics did a good job of estimating the epidemic curve in future years for some seasons but not so well for others (for the pre-existing immunity scalar value of 0.5 see Figs. 2 and 3 and for 0.25 see Figs. S4 and S5).

A comparison of the observed age-based clinical attack rates based on ILI data and the simulated rates showed better qualitative match under the specific strain infectiousness profile (Fig. S3). These rates did not vary very much and, therefore, were presented as the average values from the data. Note that our purpose in presenting the observed data was to show that qualitatively they were appropriate for the selected transmission coefficients in the simulated epidemics under each infectiousness profile and assumptions about pre-existing immunity. Also, our use of ILI data may not be as reliable as seroprevalence surveys for which we did not have any data available for our study setting. We also provided age-based clinical attack rates under different age categorizations (Fig. S14 and S15). These plots provided further evidence that the clinical attack rates were higher for younger individuals, lower for adults and even lower for seniors. These patterns are qualitatively comparable to what would be expected during a seasonal epidemic [13].

For the pre-existing scalar value of 0.5, the season-by-season fitted curves were presented by using static and dynamic forecasting. From these curves, the fit of the simulated epidemics was somewhat reliable for the 2001–2002 (up until half way to the peak week), 2004–2005 and 2005–2006. This was not the case for epidemics in the 1999–2000, 2002–2003 and 2003–2004 season. Due to similar results under each scalar for pre-existing immunity and for ease of discussion, we presented all results below for the scalar value of 0.5. Graphs for the value of 0.25 are provided in the supporting figures (Fig. S4–S13).

### Overall Fit

The overall fit between the observed data and static forecast showed mixed results (Figs. 2 and 3). Looking only at the observed data, we noticed remarkable differences in the shape of the seasonal epidemics. Some seasons were characterized by a bell-shaped curve while others were noticeably skewed towards the right (end week of the epidemic). Other seasons displayed sharp rise periods (2001–2002 and 2004–2005 season). These differences between seasons also translated into annual variation in epidemic metrics (Table 2).

**Figure 4:**
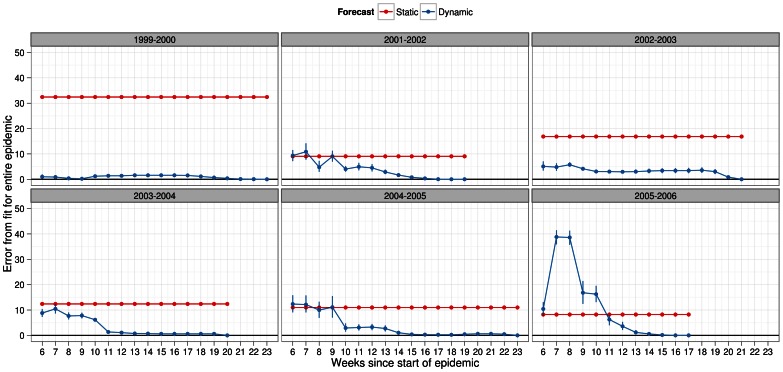
Deviation metric for overall fit in forecasts of several past influenza seasons. Overall fit was calculated under dynamic (blue) and static (red) forecasts assuming a general infectiousness profile function and scalar of 0.50 for pre-existing immunity. The metric was calculated as the % error between observed and simulated epidemics with 95% confidence intervals. For observed data we used the actual number of laboratory-confirmed samples (y-axis). In both types of forecasting, the simulated epidemic curve (scaled) was matched to the first of two consecutive weeks, in the observed epidemic, when laboratory surveillance reported 5 or more positive viral culture samples. Given our definition of the epidemic start week, this corresponded to index week 6 since the start week of the epidemic.

**Figure 5:**
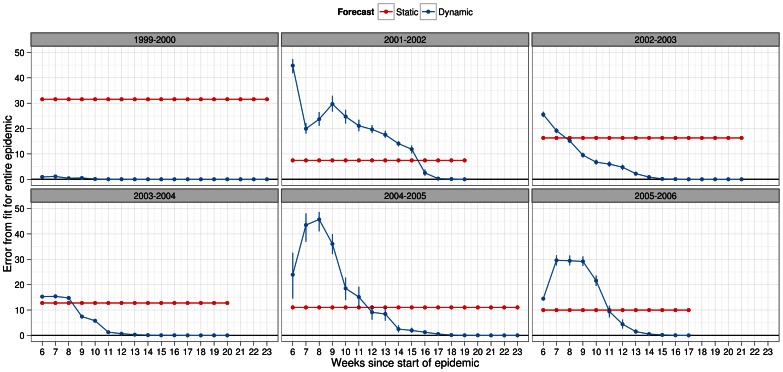
Deviation metric for overall fit in forecasts of several past influenza seasons. Overall fit was calculated under dynamic (blue) and static (red) forecasts assuming a specific infectiousness profile function and scalar of 0.50 for pre-existing immunity. The metric was calculated as the % error between observed and simulated epidemics with 95% confidence intervals. For observed data we used the actual number of laboratory-confirmed samples (y-axis). In both types of forecasting, the simulated epidemic curve (scaled) was matched to the first of two consecutive weeks, in the observed epidemic, when laboratory surveillance reported 5 or more positive viral culture samples. Given our definition of the epidemic start week, this corresponded to index week 6 since the start week of the epidemic.

In order to visualize the overall fit between the observed data and dynamic forecasts, we turned to plots of the error in overall fit for each week of prediction for each season (Figs. 4, 5, S6, S7). The purpose of these plots was to show how much better the overall fit would have been under dynamic forecasts. Under each infectiousness profile, for all seasons, the error in overall fit was at least lower by the last week of prediction in dynamic forecasts (Figs. 4–5). A main difference between the profile-based results was that 5 out of 6 seasons the fit under dynamic forecasts was always lower than static forecasts from the start of the epidemic or shortly thereafter when using the general strain profile (Fig. 4) On the other hand, this was the case in only the 2002–2003 and 2003–2004 season under the strain specific profiles (Fig. 5). In the remaining seasons, expect for the 1999–2000 season, the deviation under dynamic forecasting was below that of static forecasts more than halfway into the epidemic. In terms of the magnitude of the overall fit, we observed on average differences of about 10% between each method of forecasting except for the 1999–2000 season. In this season, we observed a special case where the simulated epidemics did a very poor job of matching the observed epidemic. This meant that the near zero error in overall fit is misleading for this seasons because the fit was just as bad using some or all of the observed data when scaling the simulated epidemic.

From these graphs, as expected, we observed more unstable estimates of the error in the overall fit at the start of the epidemic. As more weeks of prediction were used to update the forecast, these estimates stabilized and showed noticeable reductions in overall fit towards the end of the epidemic. Such stabilization occurred faster for some seasons than others and also depended on the choice of infectiousness profile function (general or strain).

### Epidemic Metrics

Epidemic metrics quantitatively characterized the spread of influenza but also provided policy-makers with meaningful measures of potential impacts. While other potential metrics were contextually relevant, such as the age-based clinical attack rate for health practitioners and vaccine coverage for public-health officials, the metrics we have used in predictive validation were more general descriptors of epidemic dynamics.

**Figure 6:**
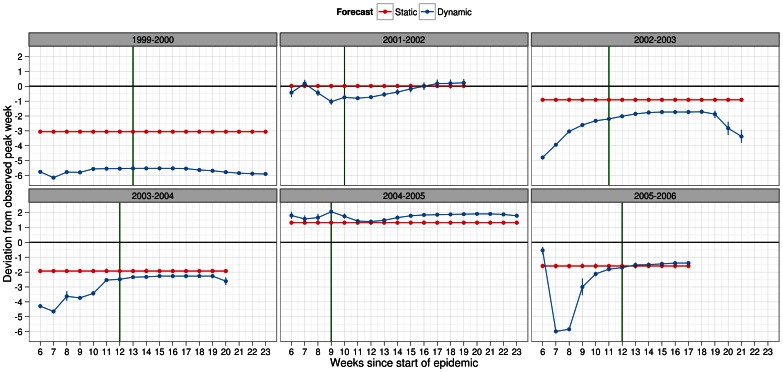
Deviation metric for peak week in forecasts of several past influenza seasons. Dynamic (blue) and static (red) forecasts were considered here assuming the general infectiousness profile function and scalar of 0.50 for pre-existing immunity. See legend of Fig. 4 for further details about x-axis. Deviation in peak week was calculated as difference in peak week of simulated and observed epidemic with 95% confidence intervals. Positive values should be interpreted as overestimation, by the forecast model, of the observed peak week. Negative values, similarly, indicated underestimation of the observed peak week. Metric values closer to zero indicated better predictive ability of the forecasting methodology. Peak week for each season (green line) was plotted to allow comparisons under each method of forecasting with regards to the timeliness of the forecast.

**Figure 7:**
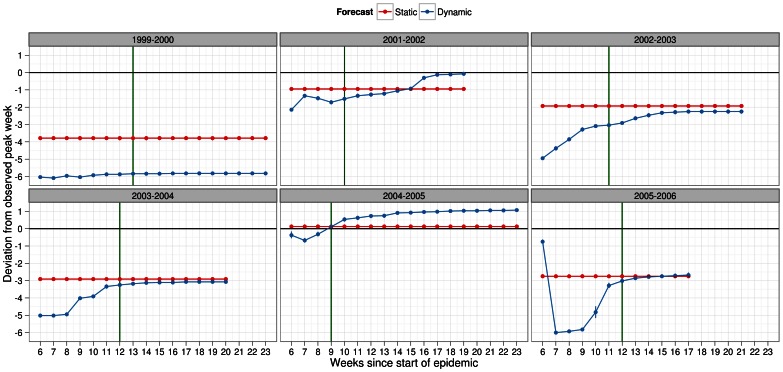
Deviation metric for peak week in forecasts of several past influenza seasons. Dynamic (blue) and static (red) forecasts were considered here assuming the specific infectiousness profile function and scalar of 0.50 for pre-existing immunity. See legend of Fig. 4 for further details about x-axis. Deviation in peak week was calculated as difference in peak week of simulated and observed epidemic with 95% confidence intervals. Positive values should be interpreted as overestimation, by the forecast model, of the observed peak week. Negative values, similarly, indicated underestimation of the observed peak week. Metric values closer to zero indicated better predictive ability of the forecasting methodology. Peak week for each season (green line) was plotted to allow comparisons under each method of forecasting with regards to the timeliness of the forecast.

Regardless of season, infectiousness profile and scaling factor for pre-existing immunity, deviations from the actual peak week were lower (i.e. closer to 0 meaning no lag from observed peak week) under static forecasting than dynamic forecasting (Fig. 6, 7, S8, S9). In terms of the magnitude, static forecasts lagged at most 3 weeks and predicted ahead at most 1 week of the observed peak week. Under dynamic forecasting, the lag may be as high as 6 week and as ahead by 2 weeks. We presented the deviations in peak week past the observed peak week to show that the reliability of dynamic forecasts did eventually improve. This likely has significant implications for which method of forecasting to use when estimating the peak week. Taking these deviations across all seasons, it was clear that static forecasts were not only much more predictive of actual peak week, but also could be made much earlier before the actual peak week of the epidemic.

**Figure 8:**
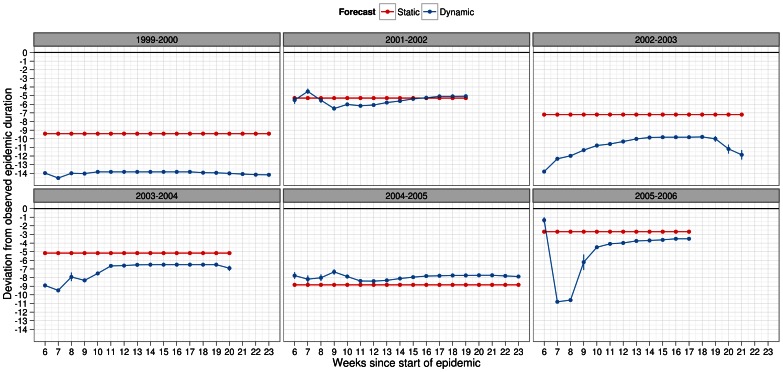
Deviation metric for epidemic duration in forecasts of several past influenza seasons. Dynamic (blue) and static (red) forecasts were considered here assuming the general infectiousness profile function and scalar of 0.50 for pre-existing immunity. See legend of Fig. 4 for further details about x-axis. Deviation in epidemic duration was calculated as difference in peak week of simulated and observed epidemic. Positive values should be interpreted as overestimation, by the forecast model, of the observed epidemic duration. Negative values, similarly, indicated underestimation of the observed epidemic duration. Metric values closer to zero indicated better predictive ability of the forecasting methodology.

**Figure 9:**
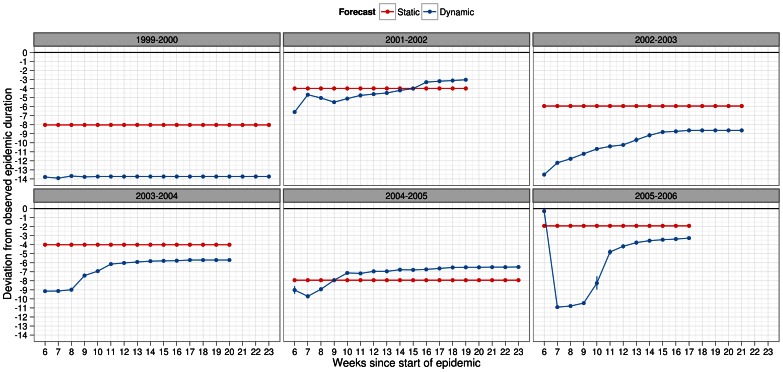
Deviation metric for epidemic duration in forecasts of several past influenza seasons. Dynamic (blue) and static (red) forecasts were considered here assuming the specific infectiousness profile function and scalar of 0.50 for pre-existing immunity. See legend of Fig. 4 for further details about x-axis. Deviation in epidemic duration was calculated as difference in peak week of simulated and observed epidemic. Positive values should be interpreted as overestimation, by the forecast model, of the observed epidemic duration. Negative values, similarly, indicated underestimation of the observed epidemic duration. Metric values closer to zero indicated better predictive ability of the forecasting methodology.

For epidemic duration, static forecasting did a better or just as good a job at forecasting the observed value across all infectiousness profiles, scalar factors for pre-existing immunity and season (Fig. 8, 9, S10, S11). Different though from peak week, both methods of forecasting showed considerable underestimation of the observed epidemic duration. In some seasons, this was as much as by 14 weeks and as little as 2 weeks. Under static forecasts, the magnitude of lag was at most 8 weeks indicating that once again this method of forecasting to be better than dynamic forecasting.

**Figure 10:**
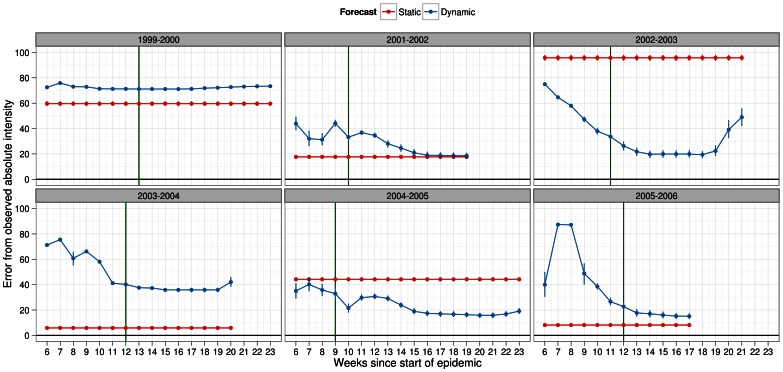
Deviation metric for absolute intensity in forecasts of several past influenza seasons. Dynamic (blue) and static (red) forecasts were considered here assuming the general infectiousness profile function and scalar of 0.50 for pre-existing immunity. See legend of Fig. 4 for details about x-axis. Absolute intensity was calculated at peak week therefore, it was redundant to estimate it after the actual peak week in the observed data. Deviation in absolute intensity was calculated as % error in absolute intensity between simulated and observed epidemic. Metric values closer to zero indicated better predictive ability of the forecasting methodology. Peak week for each season (green line) was plotted to allow comparisons under each method of forecasting with regards to the timeliness of the forecast.

**Figure 11:**
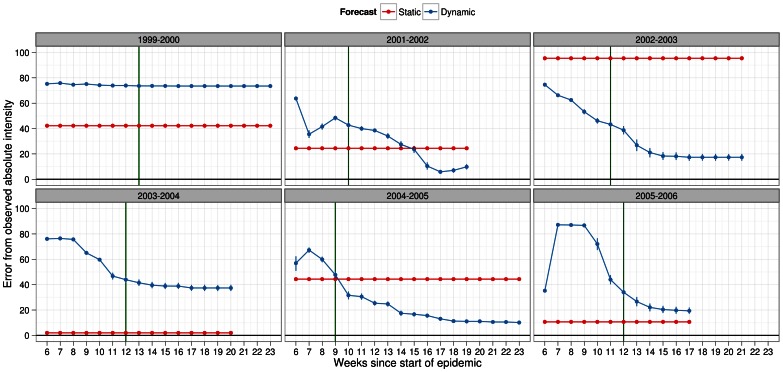
Deviation metric for absolute intensity in forecasts of several past influenza seasons. Dynamic (blue) and static (red) forecasts were considered here assuming the specific infectiousness profile function and scalar of 0.50 for pre-existing immunity. See legend of Fig. 4 for details about x-axis. Absolute intensity was calculated at peak week therefore, it was redundant to estimate it after the actual peak week in the observed data. Deviation in absolute intensity was calculated as % error in absolute intensity between simulated and observed epidemic. Metric values closer to zero indicated better predictive ability of the forecasting methodology. Peak week for each season (green line) was plotted to allow comparisons under each method of forecasting with regards to the timeliness of the forecast.

Absolute intensity was perhaps the most important epidemic metric for policy-makers as it represented the severity of the epidemic at its peak week. Better estimates of this metric as early on in the epidemic were more useful than those closer to the peak week. Our results showed that in half the seasons, we could estimate the actual absolute intensity with about 20% error or lower under static forecasting and regardless of infectiousness profile (Figs. 10 and 11). Under the same scenarios, the error in the remaining seasons was above 40% and as high as 95% for the 2002–2003 season. Under dynamic forecasting, the results were similar but tended to do a better job (i.e. lower % error) as more data was used to forecast the epidemic. The results, under the scalar factor of 0.25 for pre-existing immunity showed similar patterns (Figs. S12 and S13).

The overall message from these results indicated that it was possible to obtain reliable and timely forecasts of the observed epidemics, as measured by various epidemic metrics, under static forecasting. Another main result was that reliability and timeliness of the forecast was dependent on the epidemic metric of choice because forecasts were better for peak week and somewhat all right for absolute intensity as compared to peak week.

## Discussion

Presently national and global health agencies (e.g. CDC, WHO) invest large amounts of money and resources in influenza surveillance, forecasting and planning. The 2009 H1N1 pandemic brought to the forefront the fruits of this labor [16], [36]–[39] and demonstrates the continuing interest in using mathematical and computation models to forecast and understand the dynamics of influenza epidemics. Challenging current models of influenza spread with more rigorous definitions of prediction should provide new knowledge with practical applications for both modelers (of influenza spread) and policy-makers.

We attempt to start a conversation among policy-makers and modelers about prediction in complex models of influenza spread. Past attempts to forecast influenza epidemics have included simpler models such as temporal regression models [6], [40], [41], search engine query data models [42] and prediction market models [43]. Although these studies address real-time forecasting of influenza epidemics, they remain limited in their ability to consider some key features of interest to policy-makers. These features include spatial heterogeneity, individual heterogeneity in contact and infectiousness, and demographic, environmental and social mechanisms which affect disease transmission. As a result, more complex models were developed to explicitly consider these features through global spread models [16], [44] and individual-based models [11], [13], [14], [19]. In these complex models, simulated epidemics were fitted to a single season’s data but presented as evidence of model validation [13], [14], [32], rather than predictive model validation. Taken together, these developments show that current models for influenza epidemics with real-time forecasting ability are too simple; more complex models are useful for scenario analysis but have not been evaluated for their predictive ability. To our knowledge, this is the first study on the predictive ability of a complex model for influenza spread.

Our contribution to this discourse comes at a critical time when diverging opinions are emerging about whether prediction is even possible through mathematical models of influenza spread [40], [45], [46]. Amusingly, no study has even evaluated the predictions of complex simulation models, yet debate on the predictive ability of such models is already underway. Given the breadth of seasons and epidemic metrics in our study we go above and beyond any previous forecasting study including those with simpler models [6], [7], [47]. We specifically address two aspects of real-time forecasting of interest to policy-makers, reliability and timing. By reliability we mean the magnitude of the deviation or error in the forecast as compared to the observed data. Timing of the forecast refers to the week of prediction in which forecast becomes reliable enough to base a decision upon. In our study, we applied the process of predictive validation on a generalized version of a well-known IBM in the influenza modeling literature. We estimated epidemic metrics using forecast models which included known real-world perturbations. We showed not only how this type of validation process could be used to evaluate the reliability and timing of the forecast, but also the effect of updating the forecast on these metrics.

Ideally, policy-makers want reliable forecasts in a timely manner for each epidemic metric. Our results showed that this was certainly possible to some extent in the generalized version of the Ferguson et al. [11], [19] model. For the epidemic metric peak week and absolute intensity, reliable forecasts were possible earlier on in the epidemic under static forecasting. For the majority of the seasons, could the forecast model reliably forecasted the actual peak week with a lag of 1 to 3 weeks and, for half of all the seasons in our study, the absolute intensity with 20% error or less. No other studies using complex simulation models have been conducted to evaluate the predictive ability of the model for seasonal epidemics. Therefore, although not directly comparable, our results were better than errors of 60% or higher in forecasts of absolute intensity 1 week before actual peak week in Nishiura [7] for the 2009 H1N1 pandemic and between 20–50% error in the forecast 1 to 2 weeks before the actual peak week in Hall et al. [6] for several past pandemics. Earlier forecasts of peak week and absolute intensity provide policy-makers with several advantages with two caveats: i) forecasts were not always consistent between seasons and ii) epidemic duration was not as well predicted under static forecasting. A reason for the first caveat may be the stochastic nature of seasonal epidemics and reporting bias in our underlying observed data. For the second caveat, our methodology for forecasting, where we optimized the fit of the overall forecast rather than any one epidemic metric, may have been the reason. Instead, if our objective function was formulated to minimize the deviation between the forecast and actual peak week then under dynamic forecasting we would have expected a better fit in this metric but most likely also at the expense of higher deviations in other metrics. Therefore, an overall better fit will sometimes optimize fit for some metrics at the expense of others and this is what happened in our study.

These nuances in the method of forecasting provide important lessons for policy-makers to consider when making decisions. A lesson for policy-makers from our study was determining when to switch between static and dynamic forecasts, if at all. Static forecasts may be more reliable earlier on in an epidemic because they were actually based on data from a past epidemic and there may be uncertainty in the early phase of a present epidemic we may be trying to forecast. Later on, as more data becomes available, policy-makers could switch to dynamic forecasting since their estimates were likely to be more reliable using more data on the ongoing epidemic. Our results on the overall fit of the forecast to the observed epidemic trajectory (Figs. 4 and 5) may shed some light on this matter. In forecasts for most seasons (4 out of 6), the dynamic approach led to similar or slightly lower error in the overall fit well before the actual peak week in each season. Based on these results, we suggest the following recommendations for policy-makers. First, both static and dynamic forecasting should be used to estimate epidemic patterns in future epidemics. Second, earlier in the epidemic greater preference should be given to estimates of absolute intensity and epidemic duration under dynamic forecasts. Lastly, the reliability and timing of forecasts should be reflected in current epidemic preparedness plans through building in flexibilities for vaccine supply and stockpiling, logistical capacity for vaccine distribution and other mitigation strategies, and the efficient scheduling of resources for longer than expected epidemics. These recommendations are based solely on our results and further studies should verify these results in other geographic settings with potentially better data on past epidemics.

Our study and methodology contributes three useful insights for researchers (modelers) as well. First, clear and precise definitions of predictive validation avoid confusion between model fitting or cross validation with predictive validation [40], . Our demonstration of predictive validation hopefully relayed how it differs from current notions of prediction and the immediate benefits, in terms of reliability and timing of forecasts, it provides to policy-makers. Timely discussions of validation benefit modelers, by providing information on how to revise their models, and policy-makers, by giving them more confidence in using such models to make decisions [49]. For infectious disease modeling such discussions have only just begun [40], [45], [46]. Second, our approach greatly improves the generalizability of the model to other seasons and illustrates the flexibility of the Ferguson et al. [11], [19] model. Third, we strongly urge future modeling and predictive validation studies to consider the inclusion of perturbations in the real world. Models which ignore real-world perturbations may be limited in their ability to predict because forecasts of the future are assuming no effect of changes in the underlying mechanisms driving the epidemic process which is certainly not reflective of the real-world processes generating epidemics. There is clear evidence of this in seasonal influenza due to annual variation in vaccination coverage and the influenza strain. Furthermore, including perturbations also tests the conceptual validity of the model because the underlying processes in the model are assumed to reflect the real world. When changes in the model are not reflected in the real world then model assumptions may need to be revised accordingly.

There were several limitations of our study. In our model we did not consider delays in vaccine production and distribution, vaccine mismatch and uptake rates. We did not consider seasonal forcing variables which could potentially improve the predictive ability of the model. Potential variables included ambient temperature, crowding, co-morbidity and indoor heating [50]. It is also possible that adaptive and behavioral feedback mechanisms may affect the predictive validity of the model. These mechanisms have clear ramifications for how the epidemic trajectory will develop during the epidemic as mitigation strategies are implemented, vaccine delays are encountered and other responses to the epidemic from collective behaviors such as social distancing and school closures. Our IBM model was certainly well suited for integration of such mechanisms but it may be prudent to first assess its predictive ability in its current formulation before including additional complexity.

In conclusion, our study used a well-known complex simulation model for the spread of influenza to study its ability to forecast future epidemics. We demonstrated the process of predictive validation in the context of how model predictions are used in the real world to make forecasts about epidemic patterns before and during an epidemic event. Based on our results we provided clear and useful recommendations for policy-makers and modelers. Model validation, in general, is not an all-or-nothing affair but rather a series of statements based on applying a suite of validation techniques to a model. Since society will always desire to predict the future it would be naïve to leave out or downplay the role of predictive validation techniques in models of disease spread.

## Supporting Information

Figure S1 **Infectiousness profiles based on the Ferguson model (general strain) and Liao et al.** [**35**] **)(specific).** Curves are normalized such that area under the curve equals 1.
(TIFF)

Figure S2 **Model fitting to observed data for reference influenza season (1998–1999) under different infectiousness profile functions and scalar for pre-existing immunity set to 0.25.** Observed data are presented as the number (black solid lines) and % positive (dashed black line) of laboratory-confirmed positive samples. X-axis is the number of weeks since start of season and not calendar weeks. The start of the influenza season was defined by 5 or more positive viral cultures in two consecutive weeks. Number of laboratory-confirmed samples was used to fit the simulated epidemic curve after scaling the average number of new infections (solid blue line) to compare with observed data. We matched this scaled version of the simulated epidemic curve to the first week of the actual epidemic. 95% confidence intervals were based on 50 simulated epidemics (dotted blue lines).
(TIFF)

Figure S3 **Observed (points) and simulated (bars) age-based clinical attack rates (age categories of 0–17 and 18+) under different infectiousness profiles and scalar for pre-existing immunity.** Values were based on all available data from observed epidemics and 50 simulated epidemics with initial conditions as mentioned in the main text.
(TIFF)

Figure S4 **Forecasts of several past influenza seasons and the observed data on past epidemics (black bars, number of laboratory positive samples), assuming a general infectiousness profile function and scalar for pre-existing immunity of 0.25.** Forecasts were based on best-fit baseline model in which only the level of vaccination coverage was changed for each season. Scaling of the simulated epidemics was done under static (based on scalar for 1998–1999 season, red lines) or dynamic (based on having observed entire epidemic for respective season, blue lines). Green lines indicated %positive laboratory samples. X-axis is the number of weeks since start of season.
(TIFF)

Figure S5 **Forecasts of several past influenza seasons and the observed data on past epidemics (black bars, number of laboratory positive samples), assuming a specific infectiousness profile function and scalar for pre-existing immunity of 0.25.** Forecasts were based on best-fit baseline model in which only the level of vaccination coverage was changed for each season. Scaling of the simulated epidemics was done under static (based on scalar for 1998–1999 season, red lines) or dynamic (based on having observed entire epidemic for respective season, blue lines). Green lines indicated %positive laboratory samples. X-axis is the number of weeks since start of season.
(TIFF)

Figure S6 **Deviation metric for overall fit in forecasts of several past influenza seasons.** Overall fit was calculated under dynamic (blue) and static (red) forecasts assuming a general infectiousness profile function and scalar of 0.25 for pre-existing immunity. The metric was calculated as the % error between observed and simulated epidemics with 95% confidence intervals. For observed data we used the actual number of laboratory-confirmed samples (y-axis). In both types of forecasting, the simulated epidemic curve (scaled) was matched to the first of two consecutive weeks, in the observed epidemic, when laboratory surveillance reported 5 or more positive viral culture samples. Given our definition of the epidemic start week, this corresponded to index week 6 since the start week of the epidemic.
(TIFF)

Figure S7 **Deviation metric for overall fit in forecasts of several past influenza seasons.** Overall fit was calculated under dynamic (blue) and static (red) forecasts assuming a specific infectiousness profile function and scalar of 0.25 for pre-existing immunity. The metric was calculated as the % error between observed and simulated epidemics with 95% confidence intervals. For observed data we used the actual number of laboratory-confirmed samples (y-axis). In both types of forecasting, the simulated epidemic curve (scaled) was matched to the first of two consecutive weeks, in the observed epidemic, when laboratory surveillance reported 5 or more positive viral culture samples. Given our definition of the epidemic start week, this corresponded to index week 6 since the start week of the epidemic.
(TIFF)

Figure S8 **Deviation metric for peak week in forecasts of several past influenza seasons.** Dynamic (blue) and static (red) forecasts were considered here assuming the general infectiousness profile function and scalar of 0.25 for pre-existing immunity. See legend of Fig. 4 for further details about x-axis. Deviation in peak week was calculated as difference in peak week of simulated and observed epidemic with 95% confidence intervals. Positive values should be interpreted as overestimation, by the forecast model, of the observed peak week. Negative values, similarly, indicated underestimation of the observed peak week. Metric values closer to zero indicated better predictive ability of the forecasting methodology. Peak week for each season (green line) was plotted to allow comparisons under each method of forecasting with regards to the timeliness of the forecast.
(TIFF)

Figure S9 **Deviation metric for peak week in forecasts of several past influenza seasons.** Dynamic (blue) and static (red) forecasts were considered here assuming the specific infectiousness profile function and scalar of 0.25 for pre-existing immunity. See legend of Fig. 4 for further details about x-axis. Deviation in peak week was calculated as difference in peak week of simulated and observed epidemic with 95% confidence intervals. Positive values should be interpreted as overestimation, by the forecast model, of the observed peak week. Negative values, similarly, indicated underestimation of the observed peak week. Metric values closer to zero indicated better predictive ability of the forecasting methodology. Peak week for each season (green line) was plotted to allow comparisons under each method of forecasting with regards to the timeliness of the forecast.
(TIFF)

Figure S10 **Deviation metric for epidemic duration in forecasts of several past influenza seasons.** Dynamic (blue) and static (red) forecasts were considered here assuming the general infectiousness profile function and scalar of 0.25 for pre-existing immunity. See legend of Fig. 4 for further details about x-axis. Deviation in epidemic duration was calculated as difference in peak week of simulated and observed epidemic. Positive values should be interpreted as overestimation, by the forecast model, of the observed epidemic duration. Negative values, similarly, indicated underestimation of the observed epidemic duration. Metric values closer to zero indicated better predictive ability of the forecasting methodology.
(TIFF)

Figure S11 **Deviation metric for epidemic duration in forecasts of several past influenza seasons.** Dynamic (blue) and static (red) forecasts were considered here assuming the specific infectiousness profile function and scalar of 0.25 for pre-existing immunity. See legend of Fig. 4 for further details about x-axis. Deviation in epidemic duration was calculated as difference in peak week of simulated and observed epidemic. Positive values should be interpreted as overestimation, by the forecast model, of the observed epidemic duration. Negative values, similarly, indicated underestimation of the observed epidemic duration. Metric values closer to zero indicated better predictive ability of the forecasting methodology.
(TIFF)

Figure S12 **Deviation metric for absolute intensity in forecasts of several past influenza seasons.** Dynamic (blue) and static (red) forecasts were considered here assuming the general infectiousness profile function and scalar of 0.25 for pre-existing immunity. See legend of Fig. 4 for details about x-axis. Absolute intensity was calculated at peak week therefore, it was redundant to estimate it after the actual peak week in the observed data. Deviation in absolute intensity was calculated as % error in absolute intensity between simulated and observed epidemic. Metric values closer to zero indicated better predictive ability of the forecasting methodology. Peak week for each season (green line) was plotted to allow comparisons under each method of forecasting with regards to the timeliness of the forecast.
(TIFF)

Figure S13 **Deviation metric for absolute intensity in forecasts of several past influenza seasons.** Dynamic (blue) and static (red) forecasts were considered here assuming the specific infectiousness profile function and scalar of 0.25 for pre-existing immunity. See legend of Fig. 4 for details about x-axis. Absolute intensity was calculated at peak week therefore, it was redundant to estimate it after the actual peak week in the observed data. Deviation in absolute intensity was calculated as % error in absolute intensity between simulated and observed epidemic. Metric values closer to zero indicated better predictive ability of the forecasting methodology. Peak week for each season (green line) was plotted to allow comparisons under each method of forecasting with regards to the timeliness of the forecast.
(TIFF)

Figure S14 **Simulated age-based clinical attack rates (age categories of 0–100 in 5-year intervals) under different infectiousness profiles (panels) and scalar for pre-existing immunity (colored bars).** Values were based on all available data from observed epidemics and 50 simulated epidemics with initial conditions as mentioned in the main text.
(TIFF)

Figure S15 **Simulated age-based clinical attack rates (age categories of 0–4, 5–10, 11–20 and 20+) under different infectiousness profiles (panels) and scalar for pre-existing immunity (colored bars).** Values were based on all available data from observed epidemics and 50 simulated epidemics with initial conditions as mentioned in the main text.
(TIFF)

Text S1 **Supplementary material.**
(DOC)
