# Supplementary figures and images for: Predictive Validation of an Influenza Spread Model
Source: PLoS One. 2013 Jun 3;8(6):e65459. doi: 10.1371/journal.pone.0065459 (PMC3670880; doi:10.1371/journal.pone.0065459)

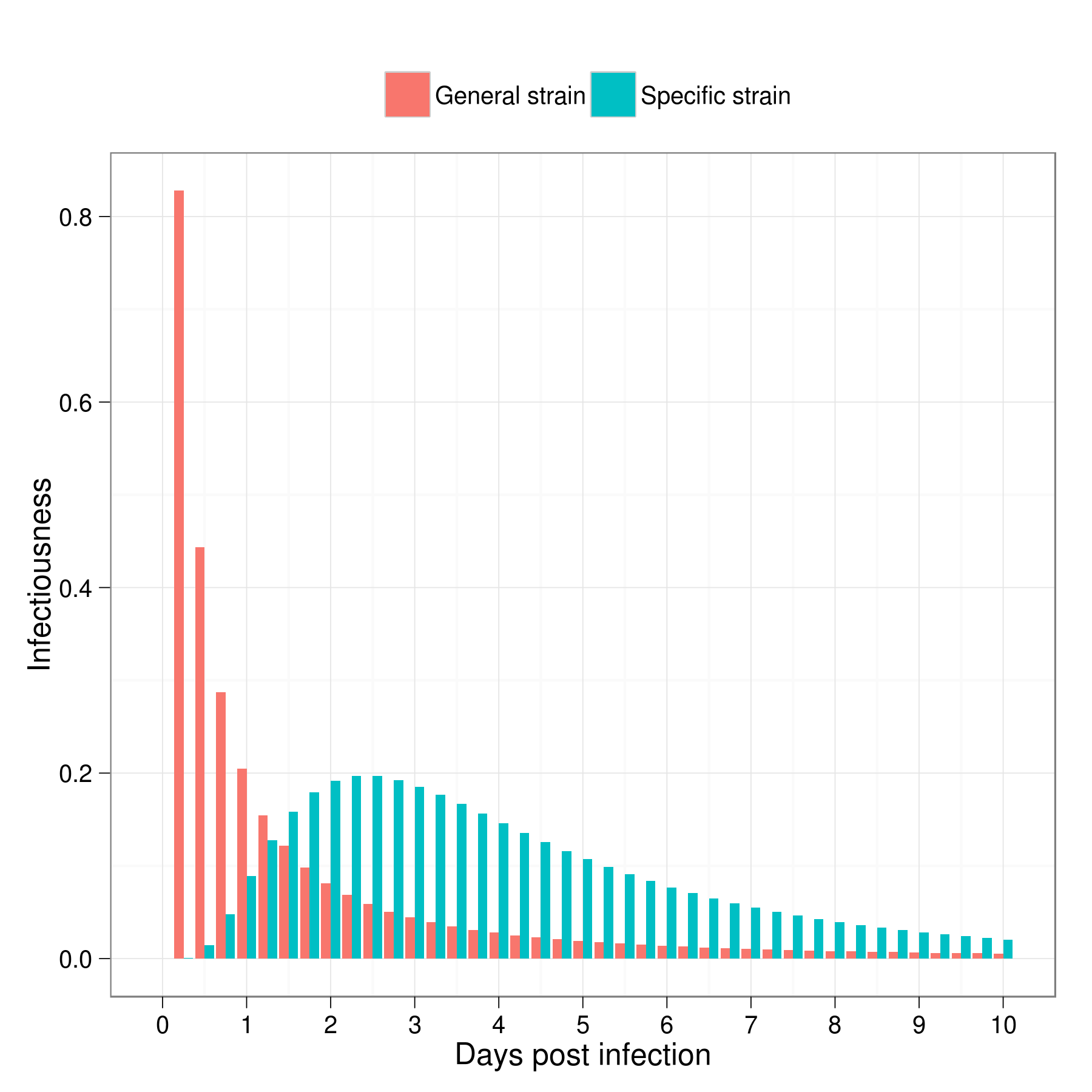

Supplement: Figure S1 — Infectiousness profiles based on the Ferguson model (general strain) and Liao et al. [35] )(specific). Curves are normalized such that area under the curve equals 1. (TIFF) [file pone.0065459.s001.tif]

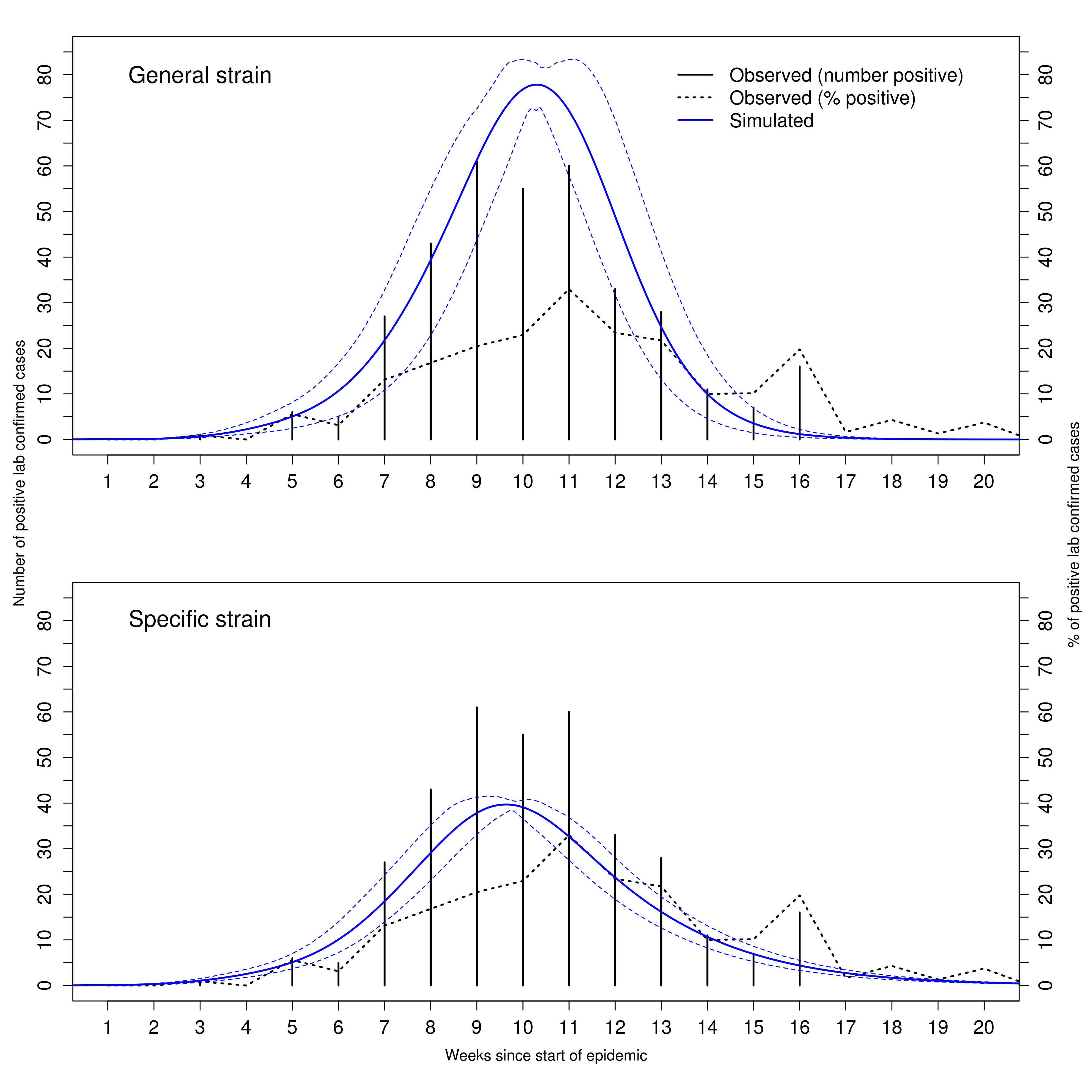

Supplement: Figure S2 — Model fitting to observed data for reference influenza season (1998–1999) under different infectiousness profile functions and scalar for pre-existing immunity set to 0.25. Observed data are presented as the number (black solid lines) and % positive (dashed black line) of laboratory-confirmed positive samples. X-axis is the number of weeks since start of season and not calendar weeks. The start of the influenza season was defined by 5 or more positive viral cultures in two consecutive weeks. Number of laboratory-confirmed samples was used to fit the simulated epidemic curve after scaling the average number of new infections (solid blue line) to compare with observed data. We matched this scaled version of the simulated epidemic curve to the first week of the actual epidemic. 95% confidence intervals were based on 50 simulated epidemics (dotted blue lines). (TIFF) [file pone.0065459.s002.tif]

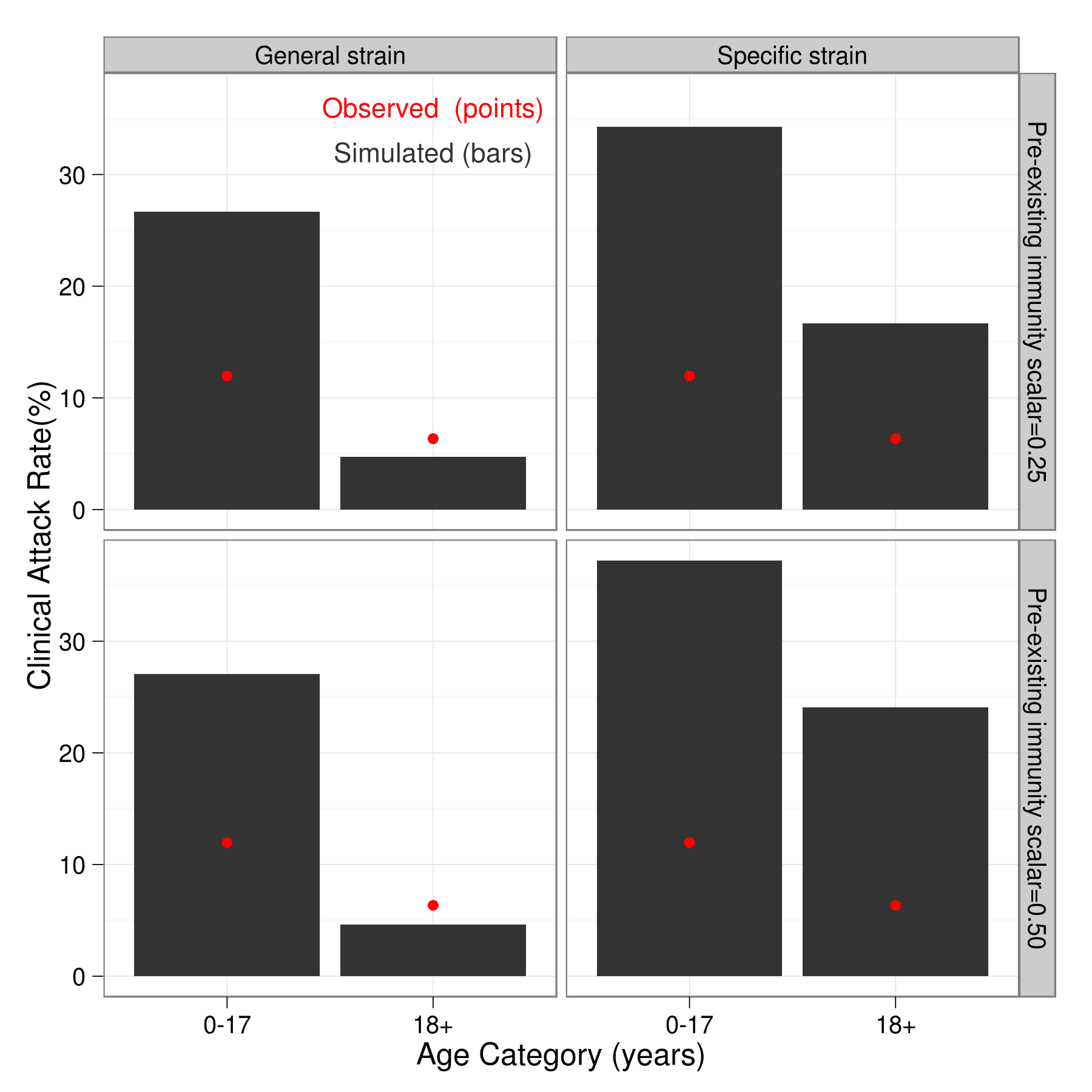

Supplement: Figure S3 — Observed (points) and simulated (bars) age-based clinical attack rates (age categories of 0–17 and 18+) under different infectiousness profiles and scalar for pre-existing immunity. Values were based on all available data from observed epidemics and 50 simulated epidemics with initial conditions as mentioned in the main text. (TIFF) [file pone.0065459.s003.tif]

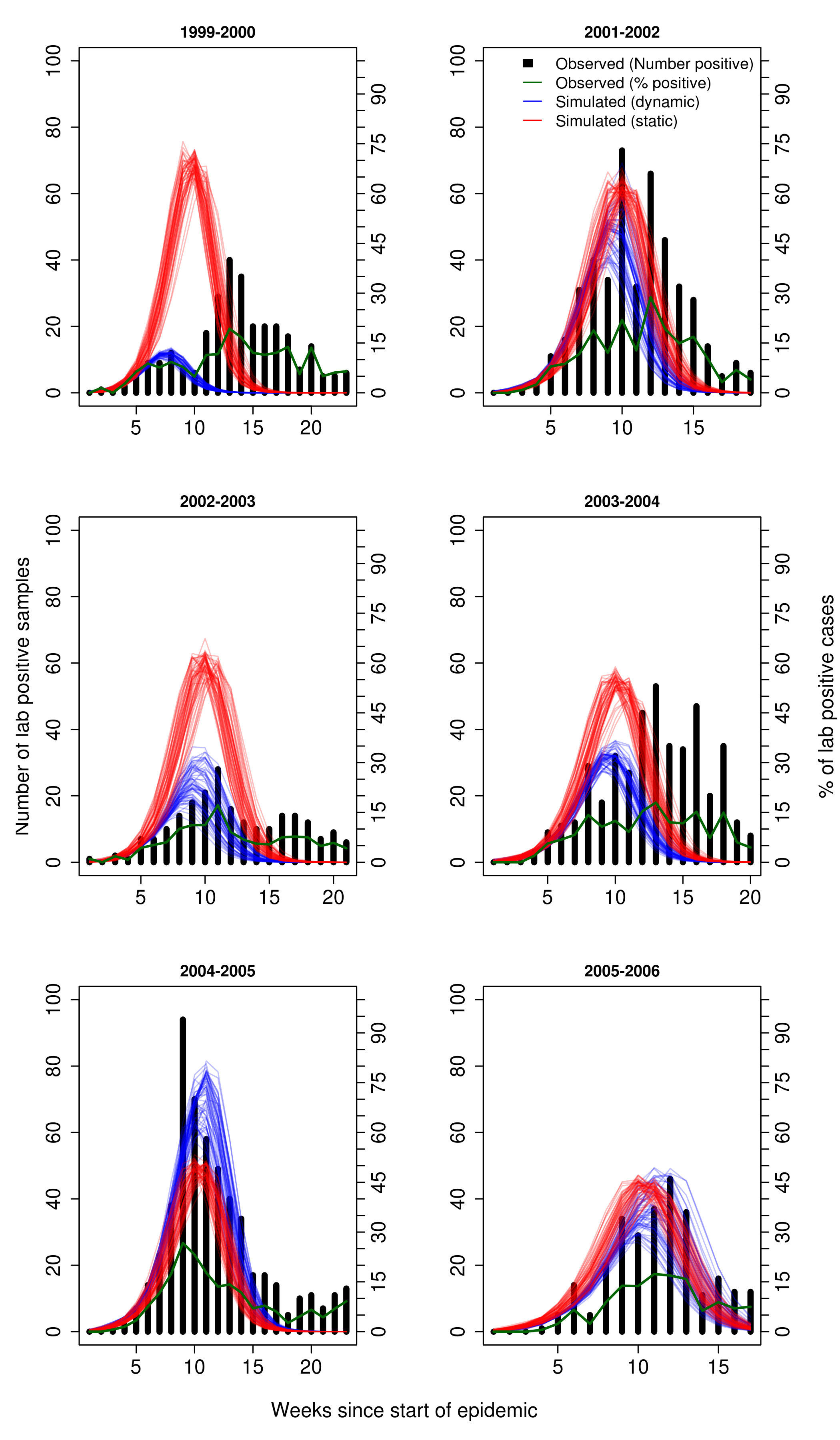

Supplement: Figure S4 — Forecasts of several past influenza seasons and the observed data on past epidemics (black bars, number of laboratory positive samples), assuming a general infectiousness profile function and scalar for pre-existing immunity of 0.25. Forecasts were based on best-fit baseline model in which only the level of vaccination coverage was changed for each season. Scaling of the simulated epidemics was done under static (based on scalar for 1998–1999 season, red lines) or dynamic (based on having observed entire epidemic for respective season, blue lines). Green lines indicated %positive laboratory samples. X-axis is the number of weeks since start of season. (TIFF) [file pone.0065459.s004.tif]

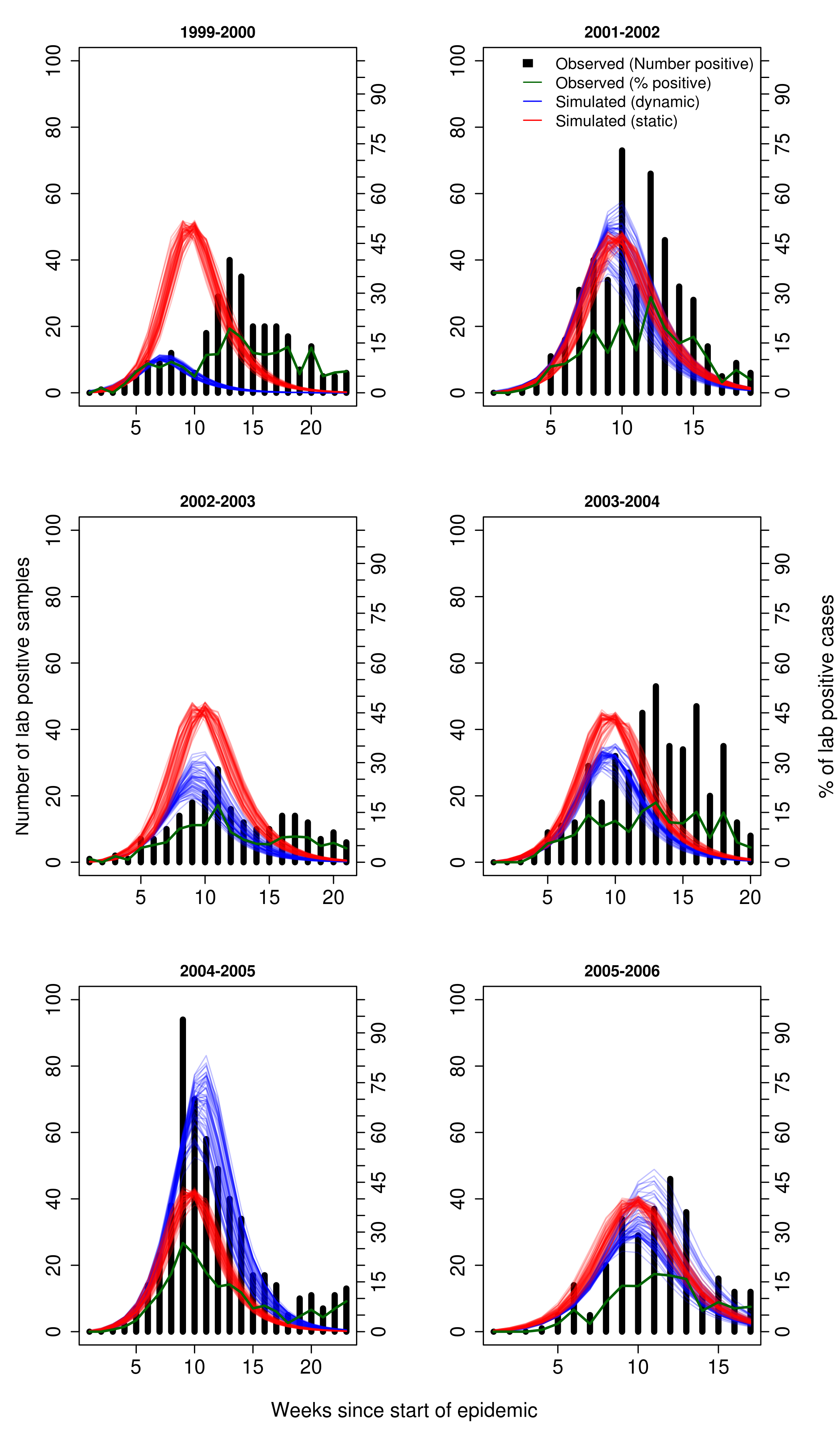

Supplement: Figure S5 — Forecasts of several past influenza seasons and the observed data on past epidemics (black bars, number of laboratory positive samples), assuming a specific infectiousness profile function and scalar for pre-existing immunity of 0.25. Forecasts were based on best-fit baseline model in which only the level of vaccination coverage was changed for each season. Scaling of the simulated epidemics was done under static (based on scalar for 1998–1999 season, red lines) or dynamic (based on having observed entire epidemic for respective season, blue lines). Green lines indicated %positive laboratory samples. X-axis is the number of weeks since start of season. (TIFF) [file pone.0065459.s005.tif]

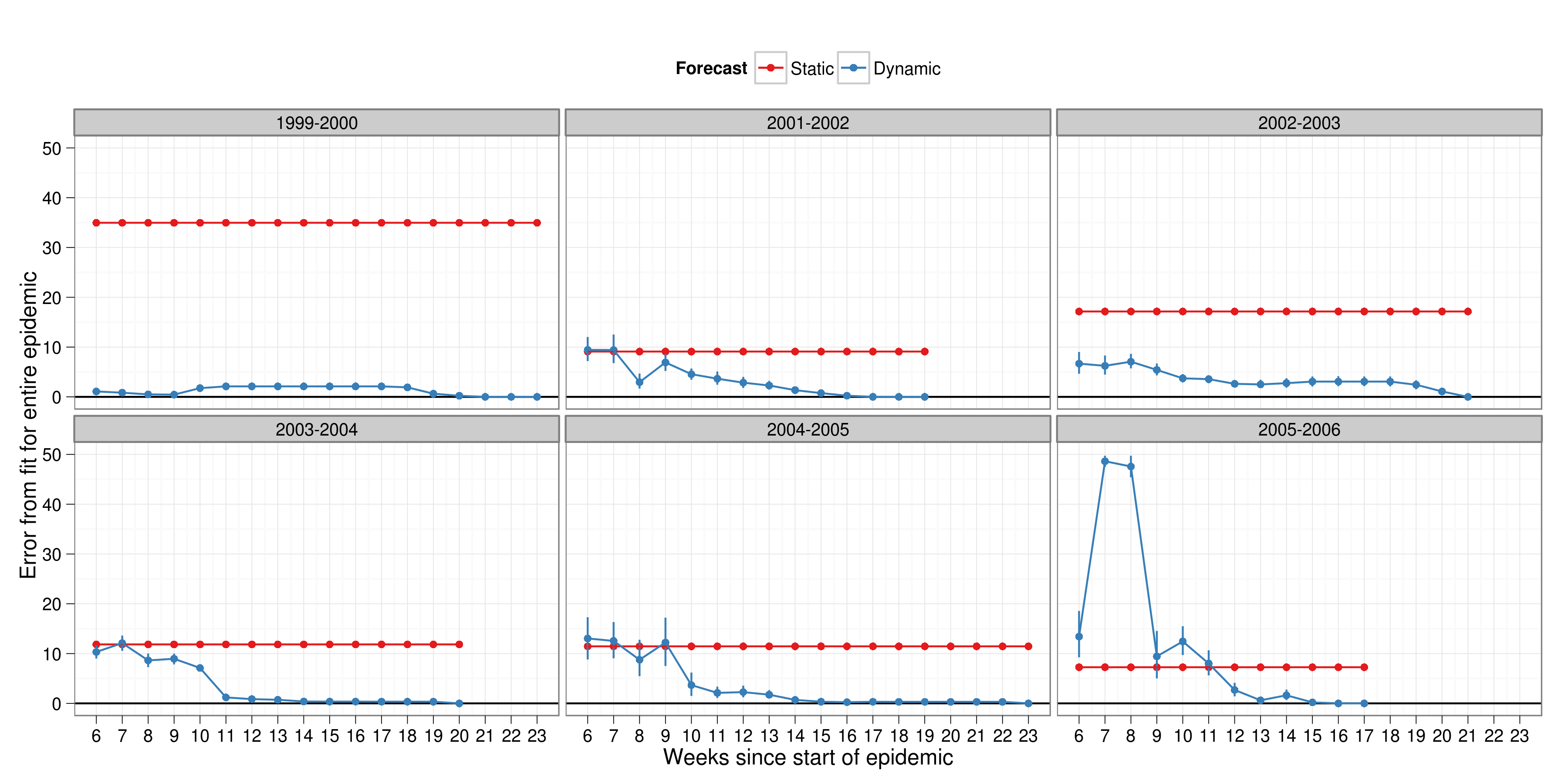

Supplement: Figure S6 — Deviation metric for overall fit in forecasts of several past influenza seasons. Overall fit was calculated under dynamic (blue) and static (red) forecasts assuming a general infectiousness profile function and scalar of 0.25 for pre-existing immunity. The metric was calculated as the % error between observed and simulated epidemics with 95% confidence intervals. For observed data we used the actual number of laboratory-confirmed samples (y-axis). In both types of forecasting, the simulated epidemic curve (scaled) was matched to the first of two consecutive weeks, in the observed epidemic, when laboratory surveillance reported 5 or more positive viral culture samples. Given our definition of the epidemic start week, this corresponded to index week 6 since the start week of the epidemic. (TIFF) [file pone.0065459.s006.tif]

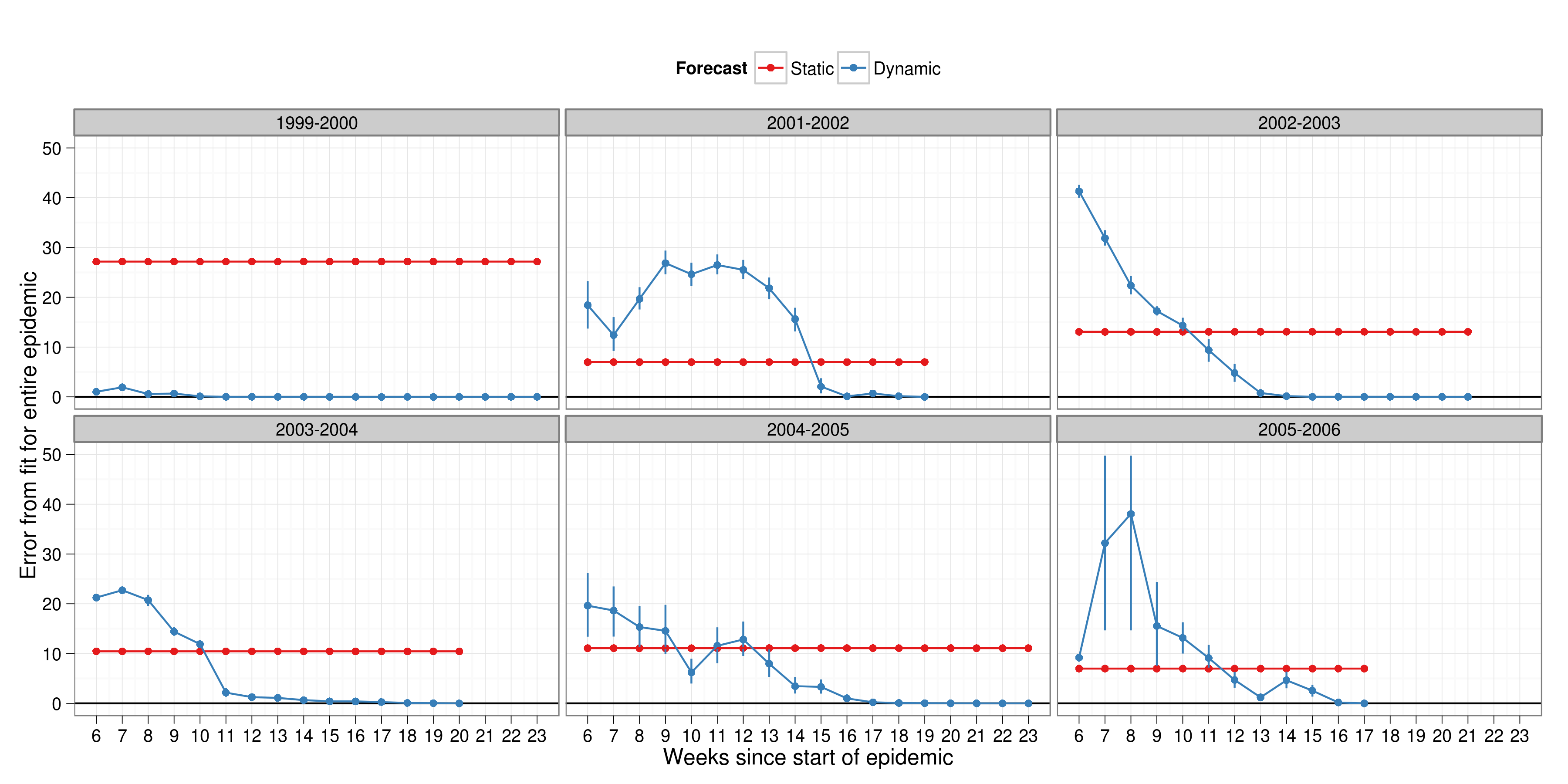

Supplement: Figure S7 — Deviation metric for overall fit in forecasts of several past influenza seasons. Overall fit was calculated under dynamic (blue) and static (red) forecasts assuming a specific infectiousness profile function and scalar of 0.25 for pre-existing immunity. The metric was calculated as the % error between observed and simulated epidemics with 95% confidence intervals. For observed data we used the actual number of laboratory-confirmed samples (y-axis). In both types of forecasting, the simulated epidemic curve (scaled) was matched to the first of two consecutive weeks, in the observed epidemic, when laboratory surveillance reported 5 or more positive viral culture samples. Given our definition of the epidemic start week, this corresponded to index week 6 since the start week of the epidemic. (TIFF) [file pone.0065459.s007.tif]

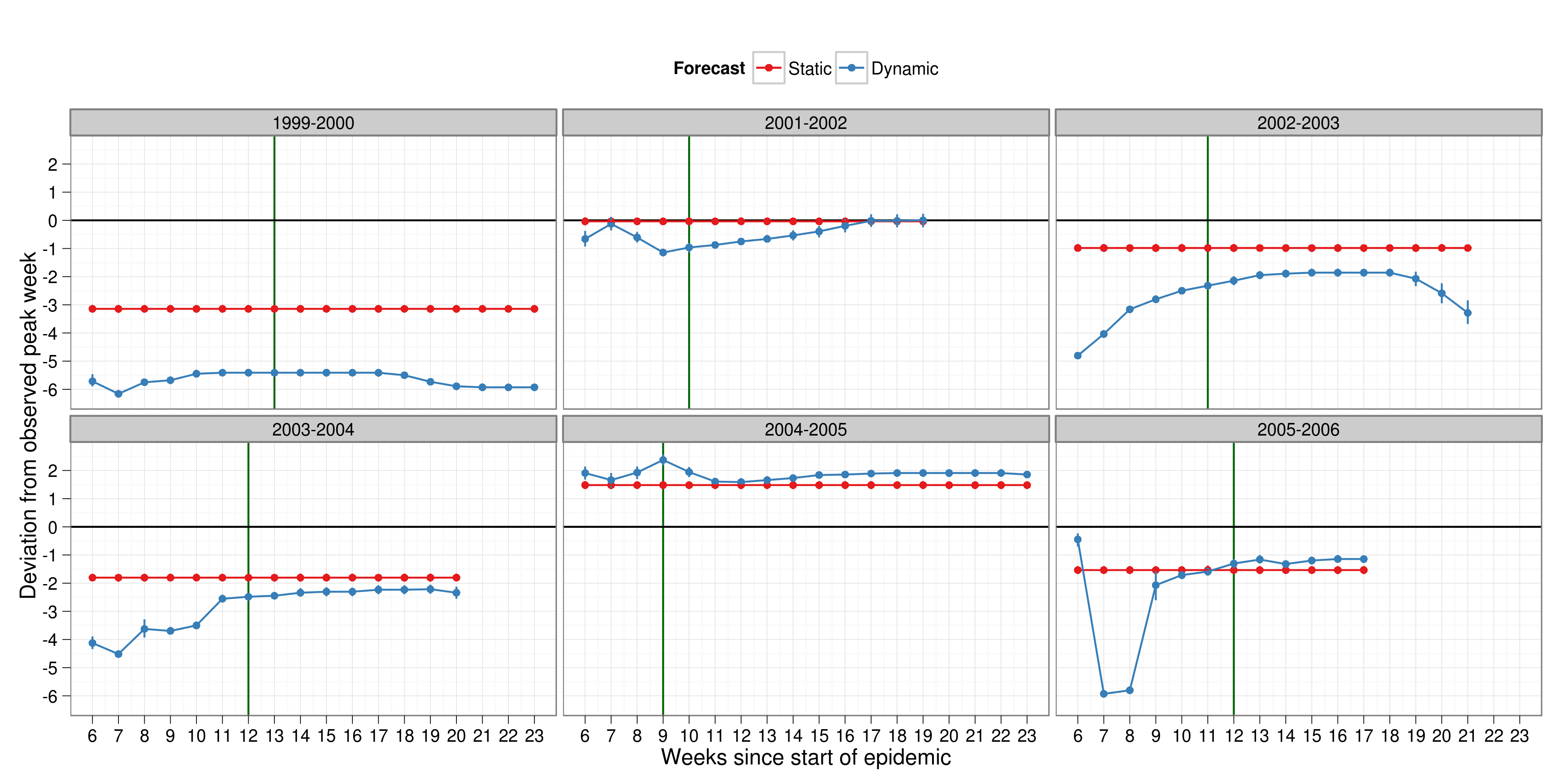

Supplement: Figure S8 — Deviation metric for peak week in forecasts of several past influenza seasons. Dynamic (blue) and static (red) forecasts were considered here assuming the general infectiousness profile function and scalar of 0.25 for pre-existing immunity. See legend of Fig. 4 for further details about x-axis. Deviation in peak week was calculated as difference in peak week of simulated and observed epidemic with 95% confidence intervals. Positive values should be interpreted as overestimation, by the forecast model, of the observed peak week. Negative values, similarly, indicated underestimation of the observed peak week. Metric values closer to zero indicated better predictive ability of the forecasting methodology. Peak week for each season (green line) was plotted to allow comparisons under each method of forecasting with regards to the timeliness of the forecast. (TIFF) [file pone.0065459.s008.tif]

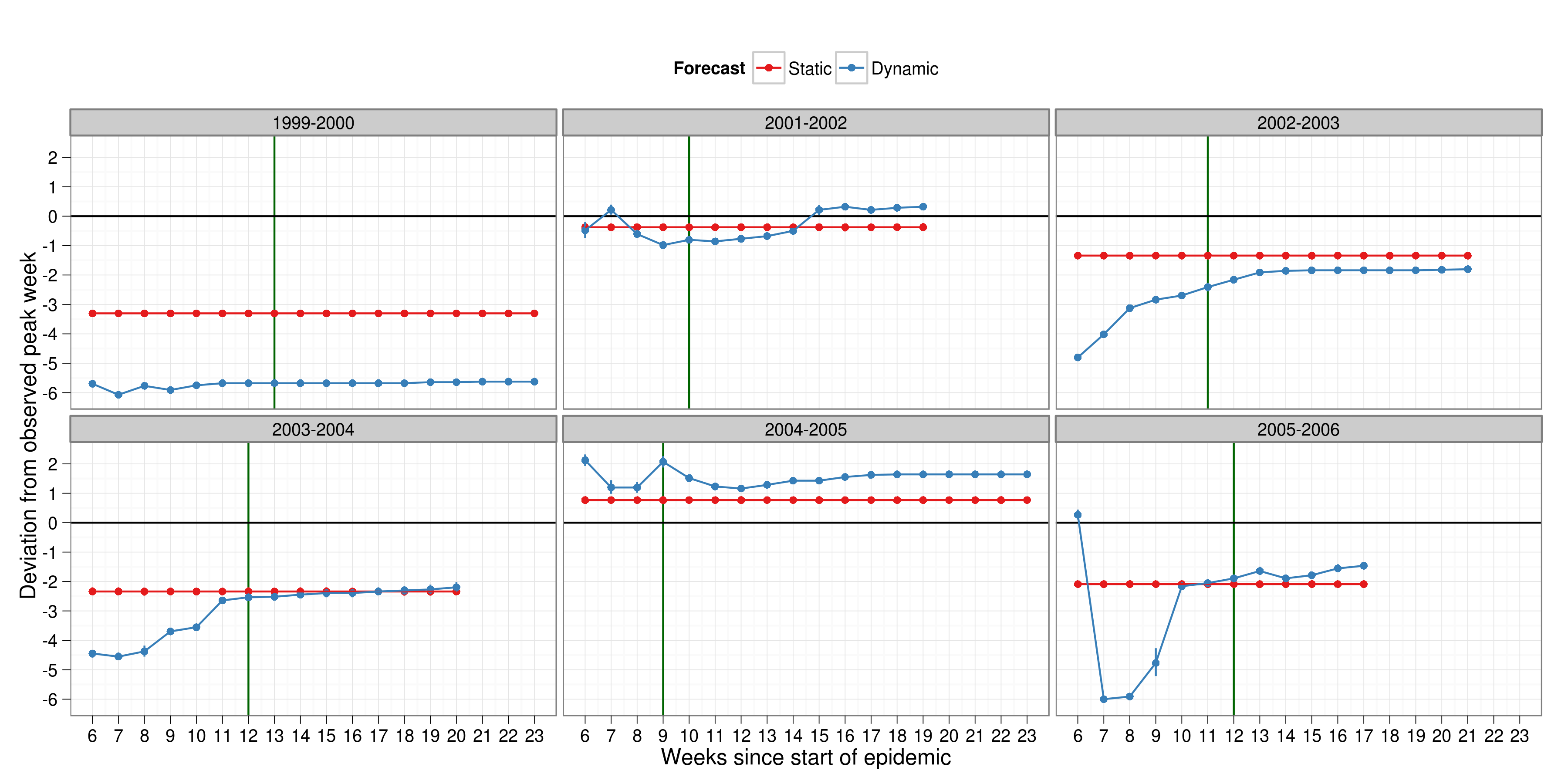

Supplement: Figure S9 — Deviation metric for peak week in forecasts of several past influenza seasons. Dynamic (blue) and static (red) forecasts were considered here assuming the specific infectiousness profile function and scalar of 0.25 for pre-existing immunity. See legend of Fig. 4 for further details about x-axis. Deviation in peak week was calculated as difference in peak week of simulated and observed epidemic with 95% confidence intervals. Positive values should be interpreted as overestimation, by the forecast model, of the observed peak week. Negative values, similarly, indicated underestimation of the observed peak week. Metric values closer to zero indicated better predictive ability of the forecasting methodology. Peak week for each season (green line) was plotted to allow comparisons under each method of forecasting with regards to the timeliness of the forecast. (TIFF) [file pone.0065459.s009.tif]

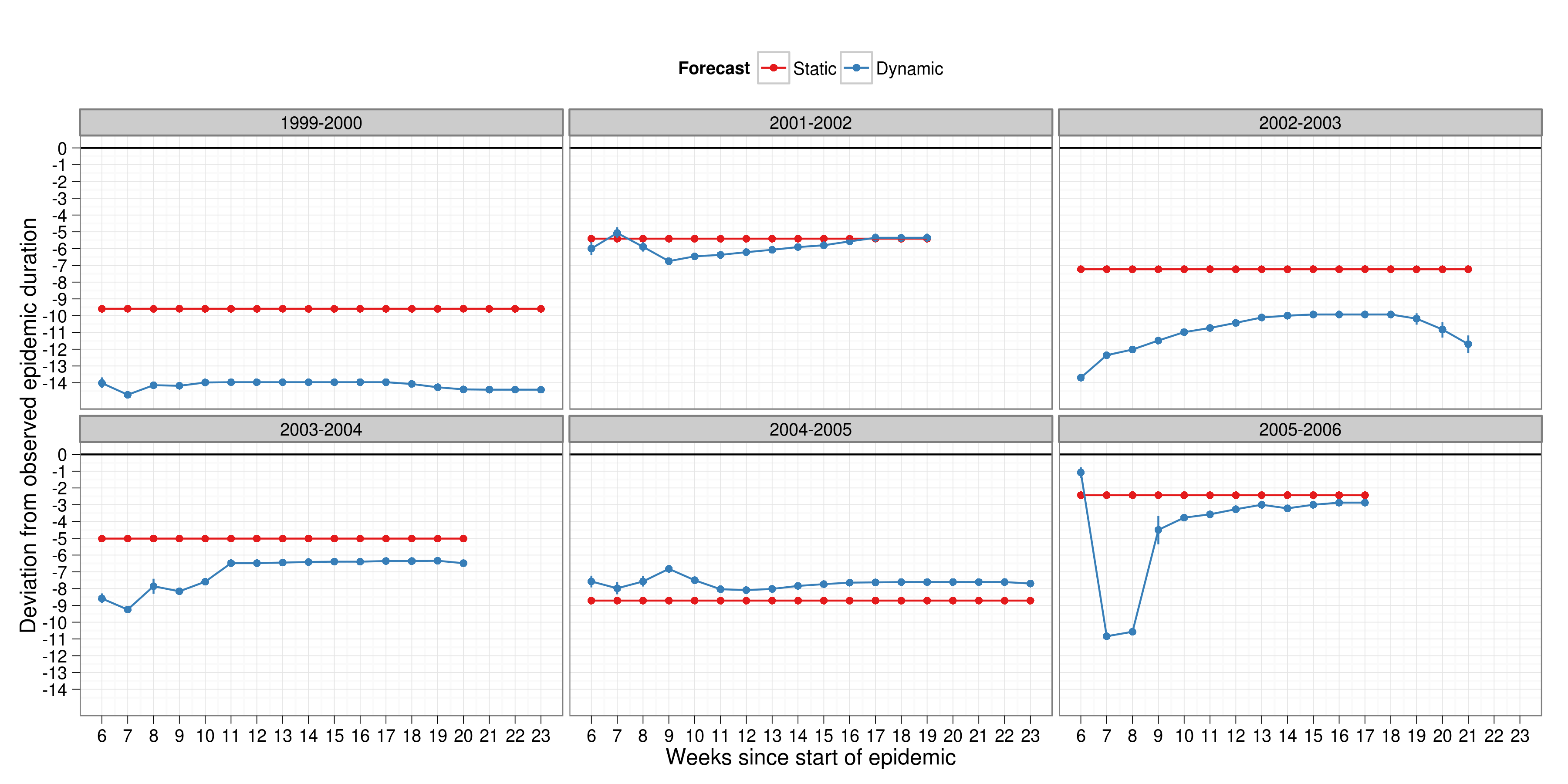

Supplement: Figure S10 — Deviation metric for epidemic duration in forecasts of several past influenza seasons. Dynamic (blue) and static (red) forecasts were considered here assuming the general infectiousness profile function and scalar of 0.25 for pre-existing immunity. See legend of Fig. 4 for further details about x-axis. Deviation in epidemic duration was calculated as difference in peak week of simulated and observed epidemic. Positive values should be interpreted as overestimation, by the forecast model, of the observed epidemic duration. Negative values, similarly, indicated underestimation of the observed epidemic duration. Metric values closer to zero indicated better predictive ability of the forecasting methodology. (TIFF) [file pone.0065459.s010.tif]

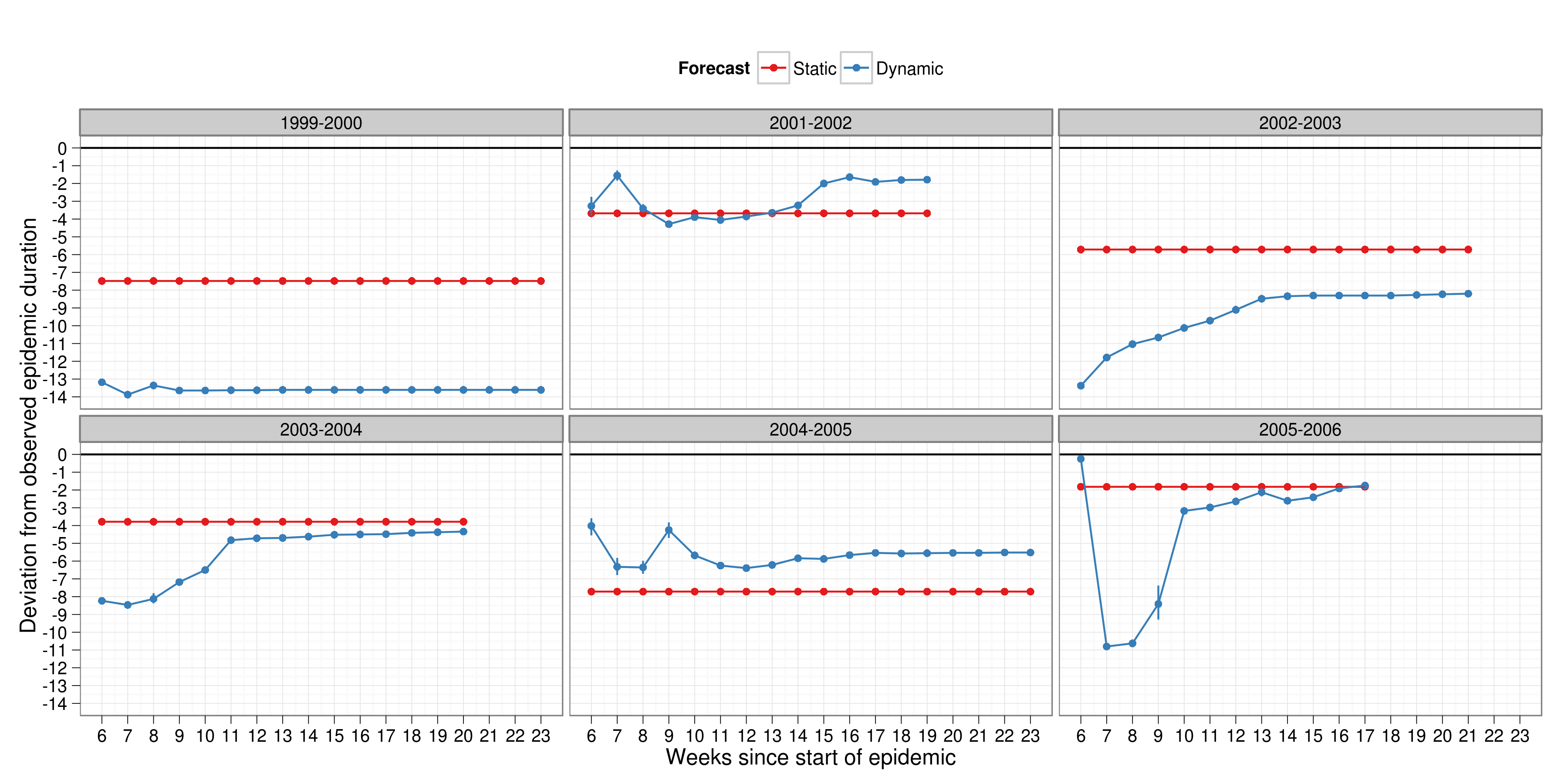

Supplement: Figure S11 — Deviation metric for epidemic duration in forecasts of several past influenza seasons. Dynamic (blue) and static (red) forecasts were considered here assuming the specific infectiousness profile function and scalar of 0.25 for pre-existing immunity. See legend of Fig. 4 for further details about x-axis. Deviation in epidemic duration was calculated as difference in peak week of simulated and observed epidemic. Positive values should be interpreted as overestimation, by the forecast model, of the observed epidemic duration. Negative values, similarly, indicated underestimation of the observed epidemic duration. Metric values closer to zero indicated better predictive ability of the forecasting methodology. (TIFF) [file pone.0065459.s011.tif]

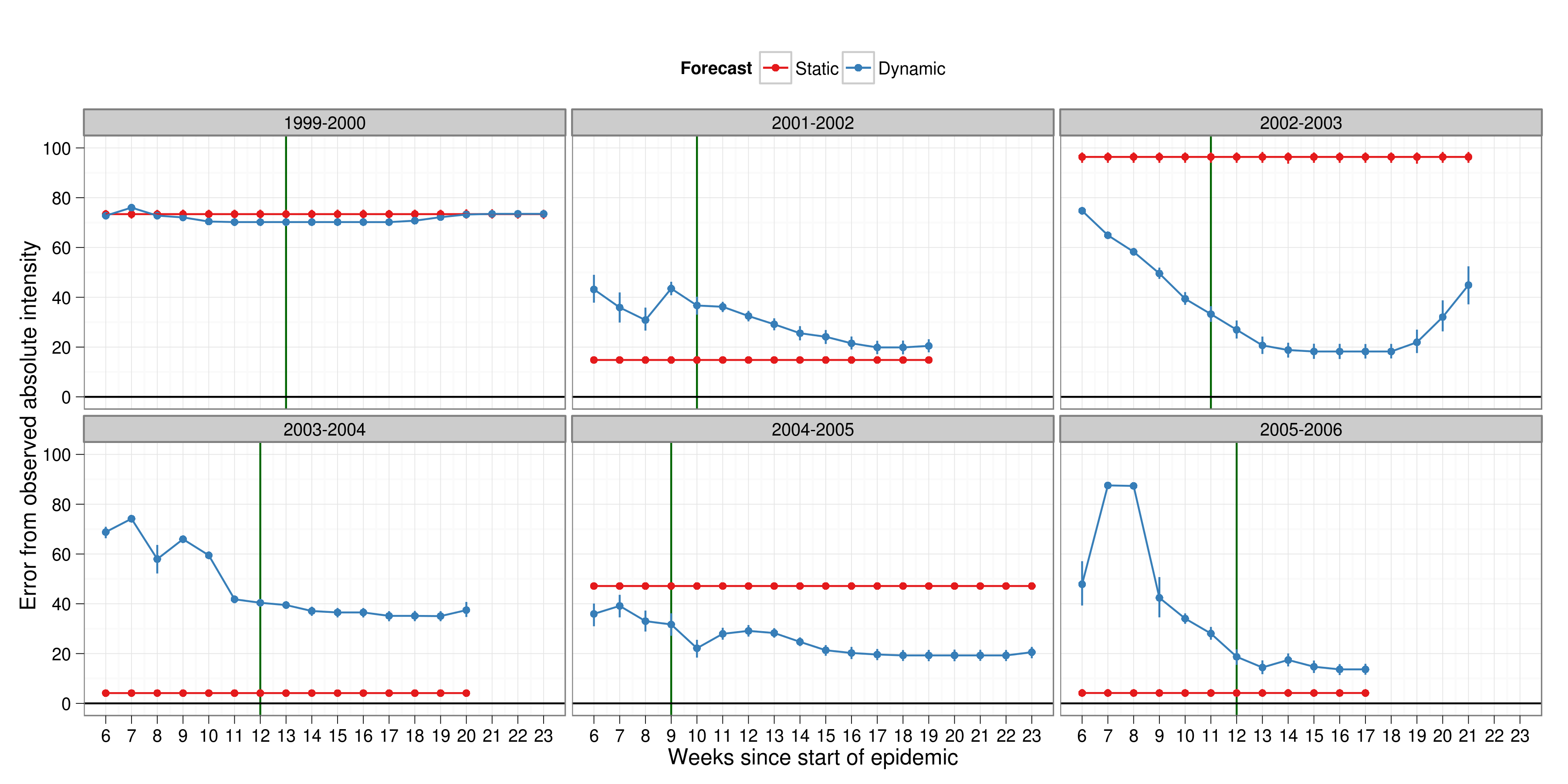

Supplement: Figure S12 — Deviation metric for absolute intensity in forecasts of several past influenza seasons. Dynamic (blue) and static (red) forecasts were considered here assuming the general infectiousness profile function and scalar of 0.25 for pre-existing immunity. See legend of Fig. 4 for details about x-axis. Absolute intensity was calculated at peak week therefore, it was redundant to estimate it after the actual peak week in the observed data. Deviation in absolute intensity was calculated as % error in absolute intensity between simulated and observed epidemic. Metric values closer to zero indicated better predictive ability of the forecasting methodology. Peak week for each season (green line) was plotted to allow comparisons under each method of forecasting with regards to the timeliness of the forecast. (TIFF) [file pone.0065459.s012.tif]

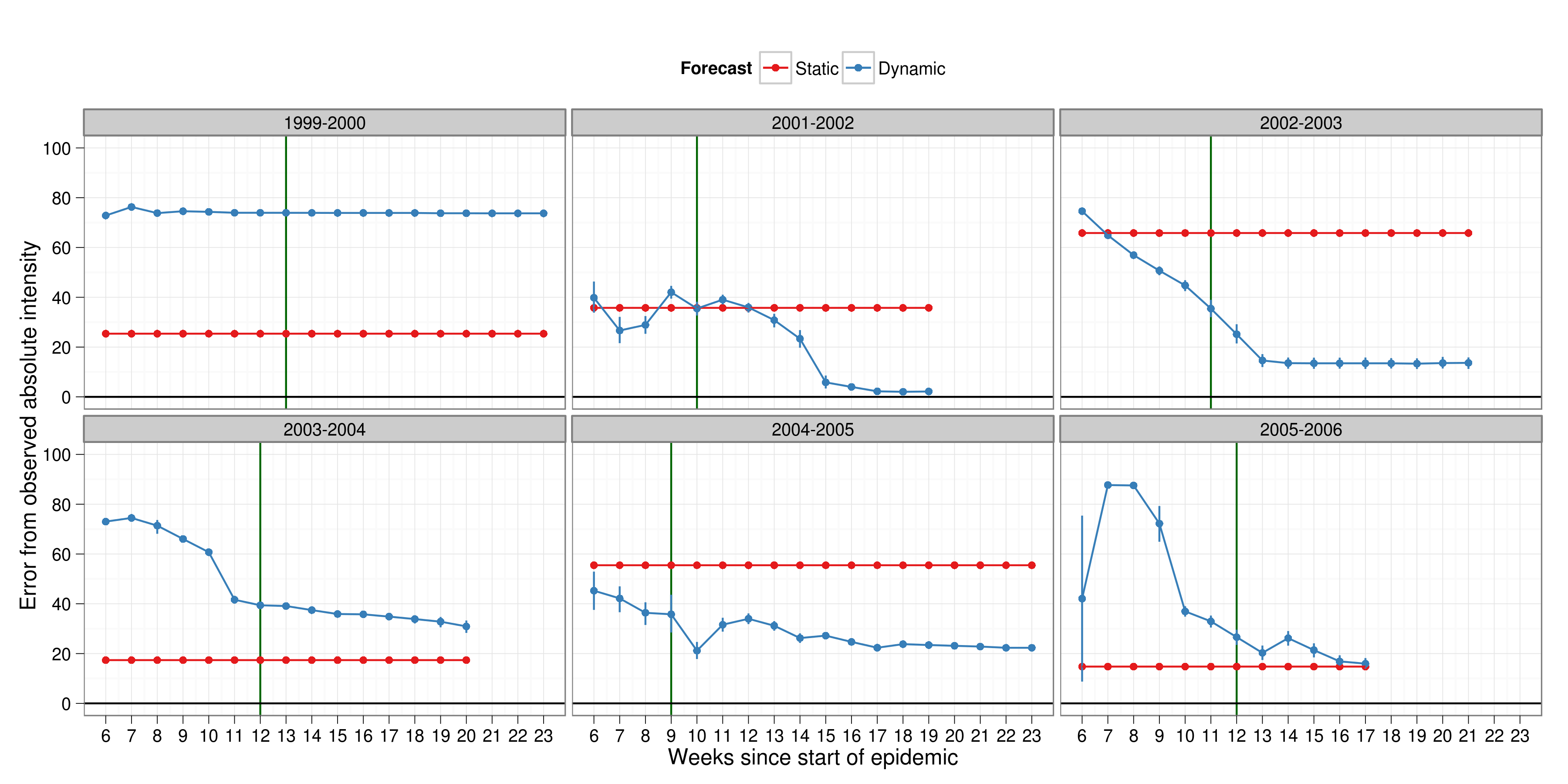

Supplement: Figure S13 — Deviation metric for absolute intensity in forecasts of several past influenza seasons. Dynamic (blue) and static (red) forecasts were considered here assuming the specific infectiousness profile function and scalar of 0.25 for pre-existing immunity. See legend of Fig. 4 for details about x-axis. Absolute intensity was calculated at peak week therefore, it was redundant to estimate it after the actual peak week in the observed data. Deviation in absolute intensity was calculated as % error in absolute intensity between simulated and observed epidemic. Metric values closer to zero indicated better predictive ability of the forecasting methodology. Peak week for each season (green line) was plotted to allow comparisons under each method of forecasting with regards to the timeliness of the forecast. (TIFF) [file pone.0065459.s013.tif]

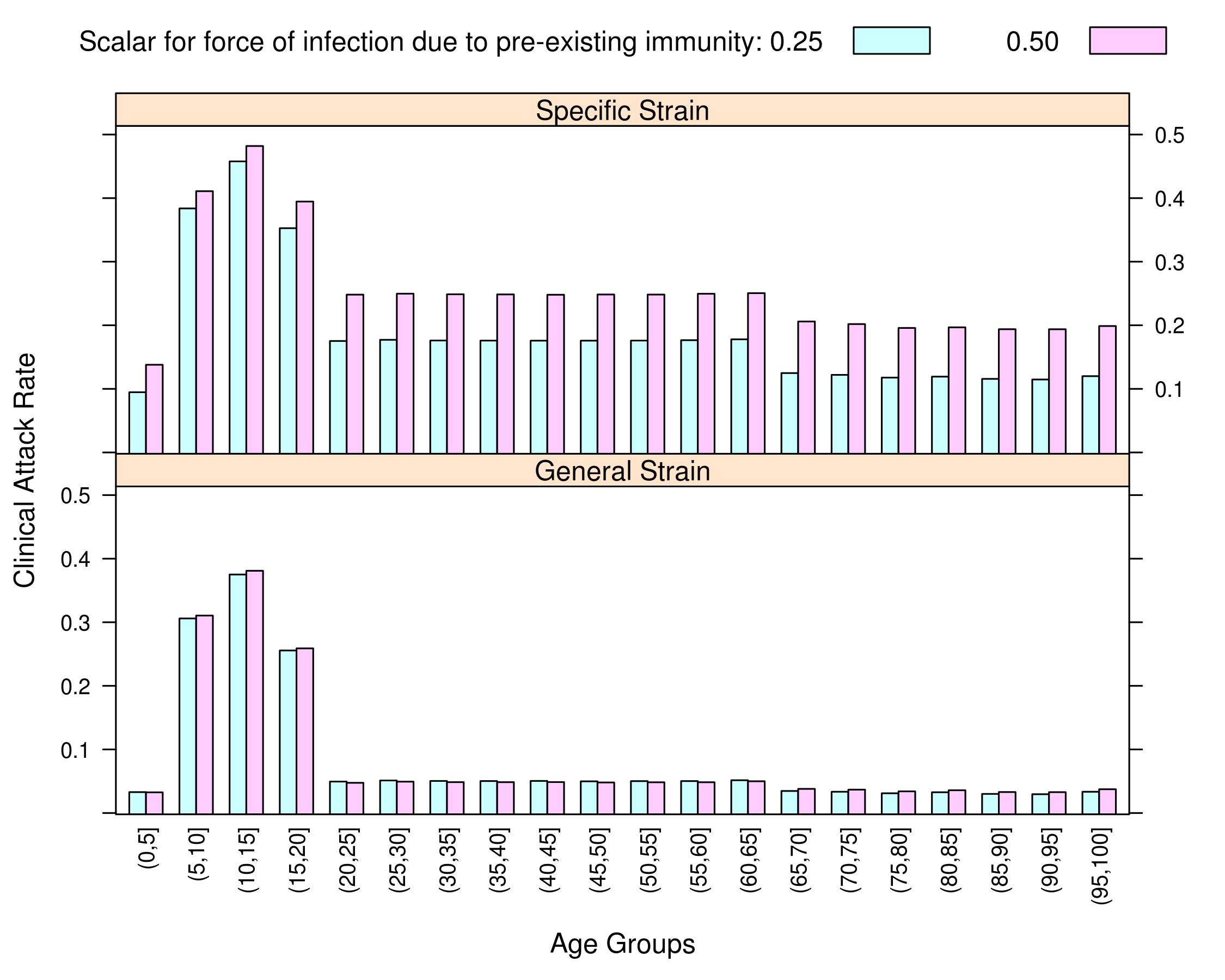

Supplement: Figure S14 — Simulated age-based clinical attack rates (age categories of 0–100 in 5-year intervals) under different infectiousness profiles (panels) and scalar for pre-existing immunity (colored bars). Values were based on all available data from observed epidemics and 50 simulated epidemics with initial conditions as mentioned in the main text. (TIFF) [file pone.0065459.s014.tif]

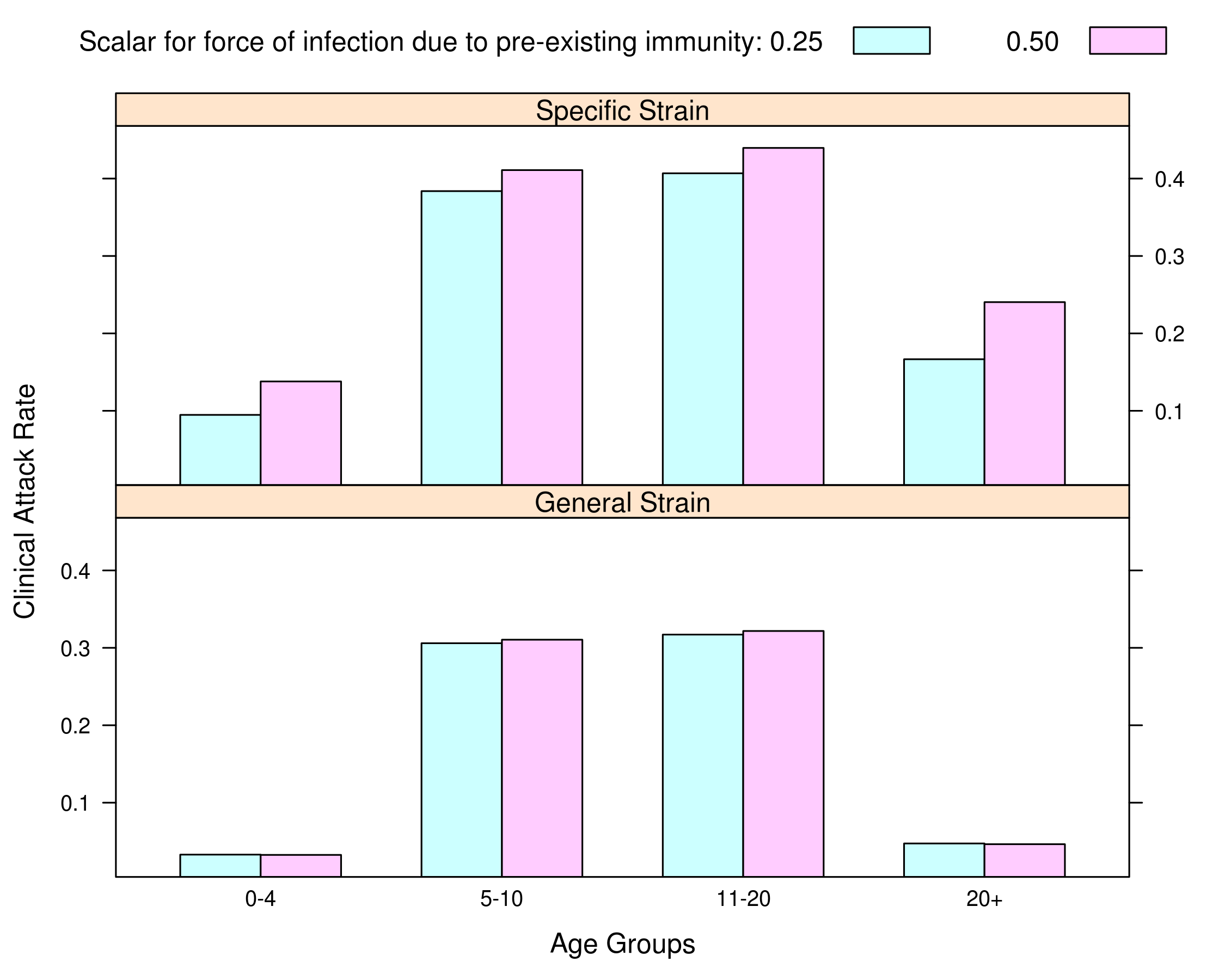

Supplement: Figure S15 — Simulated age-based clinical attack rates (age categories of 0–4, 5–10, 11–20 and 20+) under different infectiousness profiles (panels) and scalar for pre-existing immunity (colored bars). Values were based on all available data from observed epidemics and 50 simulated epidemics with initial conditions as mentioned in the main text. (TIFF) [file pone.0065459.s015.tif]
