# Supplementary material for: Predictive Validation of an Influenza Spread Model
Source: PLoS One. 2013 Jun 3;8(6):e65459. doi: 10.1371/journal.pone.0065459 (PMC3670880; doi:10.1371/journal.pone.0065459)
Supplement: Text S1 — Supplementary material. (DOC) [file pone.0065459.s016.doc]

# Supplementary Information Text

# Methods

## Details of the Montreal IBM model

Our study area had a population of approximately 3.6 million people in 2006. Since our empirical data spanned from 1996 to 2006, the 2001 Census [1] was a reasonable approximation for re-constructing the demographic characteristics of the virtual population as compared to using the most recent Census data or interpolating between census years. As a starting point, we followed the methodology of Ferguson et al. [2] in formulating our IBM model. Differently from Ferguson et al. [2] we used grid cells rather than continuous space to determine the spatial location of individuals since it made it easier to integrate the diverse types of data used to build the model. Similar to their previous work we used the LandScan [3] data set to determine the population density of individuals in 0.8 km by 0.8 km grid cells. The LandScan data provided us with a raster map of population density based on land-use, night-time light, satellite imagery, and census and administrative data. The total population size estimated from the LandScan data was 3.6 million people which exactly matched the estimated total population in the study area from the 2006 Census [4]. As a compromise between having good approximations for the spatial location of individuals and reliable and relevant estimates of population demographics and structure we made the following two assumptions. First, the total population size in the model was set to 3.6 million individuals. Second, we used data from the 2001 Census to replicate the appropriate demographic characteristics such as age and household size distributions of the population in the model.

## Households

We reconstructed individual-level characteristics using a variety of data sources. We used the Public Use Microdata Files-Families File (PUMF) from the 2001 Statistics Canada Census to recreate the observed household size distribution and family structure. The PUMF database contained randomly sampled families from all across Canada but we restricted our analysis to the 42,077 families who resided in Census Metropolitan Area (CMA) Montreal. The database also contained information on family size, type (married, common-law, or single parent), structure (with or without children), number of children at home and their age (as a categorical variable rather than an exact age). We used a heuristic algorithm to first create the same number of “virtual” families as in the database which matched in household size, type, structure and individual age. The age of individuals were drawn from the observed age distribution in CMA Montreal which was available from the Census data. To recreate households with individuals living alone we used the household size distribution data to calculate the number of such households after accounting for households greater than or equal to 2 people already in the families database. After creating households for both individuals living alone and families, we iterated through each grid cell and randomly picked a household to be located in it until the simulated grid cell matched the observed population size of the grid cell. In this way we recreated the household size, age and population density distribution from readily available data.

## Schools and workplaces

From the Quebec Ministry of Education (Ministère de l'Éducation, du Loisir et du Sport, MELS) we obtained data on the postal address and number of students in each grade level for all schools in the study area. Postal addresses were geocoded using a freely available web-based program. Children between the ages of 6 and 17, inclusive, were randomly allocated to the school closest to their place of residence. We used data on workplace size and commuting distance to create workplaces and allocated individuals to them in the IBM model. Data on workplaces was available from Statistics Canada as the number of workplaces in categories of workplace sizes. Data from the 2003 origin-destination survey [5] was used to parameterize a spatial kernel function which described the commuting patterns of individuals in the study area. This spatial kernel was used to assign individuals to workplaces. It was also used to model random community contact similar to Ferguson et al. [2]. The algorithm to assign individuals to workplace was designed to simultaneously match workplace size and commuting distance data. We used a spatial kernel of the form f(d)=1/[1+(d/a)^b^]. The best-fit parameters to observed commuting patterns from the origin-destination data were *a*=5.919 km and *b*=1.674 km. We did not consider the role of air travel or immigration/emigration in CMA Montreal since we were mainly concerned with local scale spread rather than regional [2] or global spread [6]. Also our study area encompassed most major urban centers near the city of Montreal. Therefore, the influence of global air travel or regional transportation may only be apparent when comparing epidemic patterns at larger spatial scales such as countries and provinces rather than at the metropolitan area spatial scale in our study.

## Mathematical details of the disease transmission process

The period of exposure (i.e. latent period) for each individual was drawn from a right-shifted Weibull distribution with shape and scale parameters of 1.48 and 0.47 days, respectively. At the end of the exposure period individuals become symptomatic and their infectiousness was described an infectiousness profile function. The form of this function was the lognormal distribution with a mean (μ) of -0.72 log(days) and standard deviations(σ) of 1.8 log(days). The period of infectiousness was truncated at 10 days. Recovered individuals were assumed to be immune or have died and, therefore, did not re-enter the susceptible state. For each source of infectious contact the transmission parameters were those estimated in the main text. Extensive details regarding the assumptions, formulation and parameterization of the disease transmission parameters may be found in Ferguson et al. [2].

We calculated the individual force of infection (λ_i_) following the methodology of Ferguson et al. [2].

$\begin{matrix} \lambda_{i}= & \begin{matrix} \sum_{k|h_{k}=h_{i}} \frac{I_{k}\beta_{h}\kappa\left(t-\tau_{k}\right)\rho_{k}\left[1+C_{k}\left(\omega-1\right)\right]}{n_{i}^{\alpha}} + \\ \sum_{j,k|l_{k}^{j}=l_{i}^{j}} \frac{I_{k}\beta_{p}^{j}\kappa\left(t-\tau_{k}\right)\rho_{k}\left[1+C_{k}\left(\omega\psi_{p}^{j}\kappa\left(t-\tau_{k}\right)-1\right)\right]}{m_{i}^{j}} + \\ \sum_{k} \frac{I_{k}\xi\left(a_{i}\right)\beta_{c}\kappa\left(t-\tau_{k}\right)\rho_{k}f\left(d_{i,k}\right)\left[1+C_{k}\left(\omega-1\right)\right]}{\sum_{k} f\left(d_{i,k}\right)} \end{matrix} \end{matrix}$ (1)

I_k_, binary state variable to indicate infected status. 0=not infected, 1=infected.

β_h_, household transmission parameter.

$\beta_{p}^{j}$, place transmission parameter. *j* denotes place type. We considered only schools (*j*=1) and workplaces (*j*=2)

β_c_ community transmission parameter

$\kappa(t-\tau_{k})$, infectiousness at time since infection $(t-\tau_{k})$ where *t* is current time and $\tau_{k}$ is the time when individual entered the infection class

$\psi_{p}^{j}(t-\tau_{k})$, factor by which within-place contacts change for symptomatic cases with severe infection (reflecting sickness-induced absenteeism) as a function of time since onset of disease symptoms (i.e. infectiousness)

$f(d_{i,k})$, spatial kernel function at distance *d_i,k_* between individuals *i* and *k* as determined by the gravity model

$\zeta(a_{i})$, relative travel-related (non-work) contact rate of an individual of age *a_i_*. Values were the same as in [7]

$\rho_{k}$, relative infectiousness of individual *k*, set to 1 in the model

$C_{k}$, binary state variable to indicate severe infection status, 0=not severe infection, 1=severe infection. 50% of infections were assumed to be severe.

ω, scalar for the relative infectiousness of a severe infection relative to a mild one. Value set to 2.

$n_{i}^{\alpha}$, household size. α, exponent which determines scaling of household transmission rate with household size. Value of α set to 0.8.

$m_{i}^{j}$, place size.

$\psi_{p}^{j}(t-\tau_{k})$ = 0.2 in schools and 0.5 in workplaces if $(t-\tau_{k})>0$

= 0 if $(t-\tau_{k})=0$

$f(d_{i,k})=\frac{1}{1+\left(\frac{d}{a}\right)^{;b}}$, where *a*=5.191 and *b*=1.674 based on origin-destination data from Montreal CMA

# References

1. Statistics Canada (2003) Census of Population, 2001. Ottawa, ON, Canada: Statistics Canada.

2. Ferguson NM, Cummings DA, Fraser C, Cajka JC, Cooley PC, et al. (2006) Strategies for mitigating an influenza pandemic. Nature 442: 448-452.

3. Oak Ridge National Laborary (2007) LandScan 2007 High Resolution global Population Data Set. In: UT-Battelle L, editor.

4. Statistics Canada (2006) Census of the population, 2006. Ottawa, ON, Canada: Statistics Canada.

5. L'Agence metropolitaine de transport (2003) L'Enquete origine-destination.

6. Colizza V, Barrat A, Barthelemy M, Valleron AJ, Vespignani A (2007) Modeling the worldwide spread of pandemic influenza: baseline case and containment interventions. PLoS Med 4: e13.

7. Ferguson NM, Cummings DAT, Cauchemez S, Fraser C, Riley S, et al. (2005) Strategies for containing an emerging influenza pandemic in Southeast Asia. Nature 437: 209-214.
